# Chiral Light Emission from a Hybrid Magnetic Molecule–Monolayer Transition Metal Dichalcogenide Heterostructure

**DOI:** 10.1021/acsnano.2c08320

**Published:** 2023-01-18

**Authors:** Vaibhav Varade, Golam Haider, Artur Slobodeniuk, Richard Korytar, Tomas Novotny, Vaclav Holy, Jiri Miksatko, Jan Plsek, Jan Sykora, Miriam Basova, Martin Zacek, Martin Hof, Martin Kalbac, Jana Vejpravova

**Affiliations:** † Department of Condensed Matter Physics, Faculty of Mathematics and Physics, Charles University, Ke Karlovu 5, 121 16Prague 2, Czech Republic; ‡ J. Heyrovsky Institute of Physical Chemistry, Dolejskova 3, 182 23Prague 8, Czech Republic

**Keywords:** Layered materials, transition metal dichalcogenides, molecular magnets, valley polarization, valley−spin hybrid materials, nonradiative energy drain mechanism

## Abstract

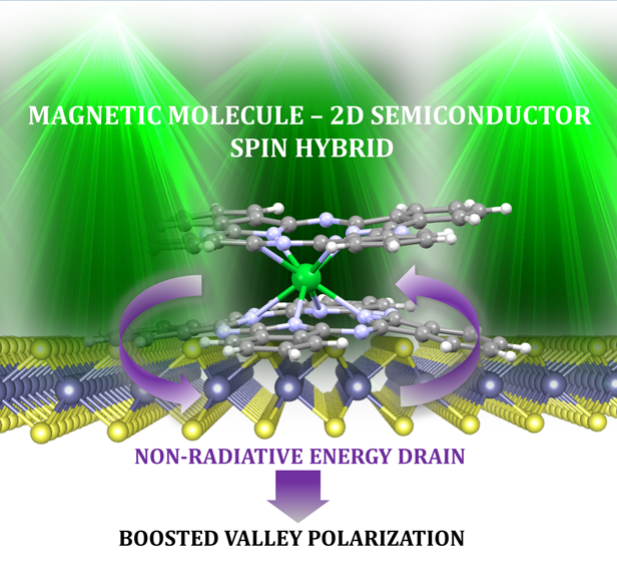

Hybrid layered materials assembled from atomically thin crystals and small molecules bring great promises in pushing the current information and quantum technologies beyond the frontiers. We demonstrate here a class of layered valley–spin hybrid (VSH) materials composed of a monolayer two-dimensional (2D) semiconductor and double-decker single molecule magnets (SMMs). We have materialized a VSH prototype by thermal evaporation of terbium bis-phthalocyanine onto a MoS_2_ monolayer and revealed its composition and stability by both microscopic and spectroscopic probes. The interaction of the VSH components gives rise to the intersystem crossing of the photogenerated carriers and moderate p-doping of the MoS_2_ monolayer, as corroborated by the density functional theory calculations. We further explored the valley contrast by helicity-resolved photoluminescence (PL) microspectroscopy carried out down to liquid helium temperatures and in the presence of the external magnetic field. The most striking feature of the VSH is the enhanced *A* exciton-related valley emission observed at the out-of-resonance condition at room temperature, which we elucidated by the proposed nonradiative energy drain transfer mechanism. Our study thus demonstrates the experimental feasibility and great promises of the ultrathin VSH materials with chiral light emission, operable by physical fields for emerging opto-spintronic, valleytronic, and quantum information concepts.

Two-dimensional (2D) materials have been an intense research focus in the past decade.^1,2^ In particular, transition metal dichalcogenides (TMDs) have emerged as a class of 2D materials with promising optical and electronic properties, which offer diverse applications, such as field-effect transistors, nanoscale optoelectronic devices (light-emitting diodes, lasers, and optical detectors) and photonic circuits.^3−6^ Moreover, mono- and double-layer TMDs are well-known for their valley-selective physics due to the Berry curvature of the Bloch band acting as an effective magnetic field that induces transverse velocity to charge carriers without the presence of external magnetic fields.^7,8^ The consequent transverse responses have been observed in all degrees of freedom of Bloch electrons, including charge, spin, and valley degeneracy, and the latter can be conveniently resolved and studied using circularly polarized (CP) light.^7,8^

One of the most explored TMDs is (1L)MoS_2_ with a hexagonal 2H crystal structure and direct band gap in the monolayer (1L) limit, which shows strong and tunable photoluminescence (PL) in the visible region.^9^ The variation in the PL of (1L)MoS_2_ is not only due to the indirect–direct band gap transition but also influenced by several other factors, including strain, doping, and defect density,^10,11^ which are strongly sample dependent and typically vary on the scale of micro- and nanometers within a single flake.^12^ Nevertheless, control of the PL via charge transfer by interfacing a TMD with another responsive species has been addressed recently.

A promising strategy is to control the PL with the help of the adsorption of different molecules or quantum dots. For example, PL tuning has been demonstrated using organic molecule 3,4,9,10-perylene tetracarboxylic dianhydride.^13^ Furthermore, a few experimental and theoretical studies suggested that modulation of the optical and electronic properties of TMDs can be achieved via charge transfer between the TMD and adsorbed π-conjugated organic molecules.^14,15^ Therefore, organic π-conjugated metal–phthalocyanines (Pc’s) are of particular interest due to their planar structure, convenient for stacking onto the TMD surface, as well as for their electronic, optical, and magnetic tunability, which are functions of the central metal. Such mixed-dimensional heterostructures have attracted reasonable interest.^16,17^

For example, it has been shown that copper phthalocyanine (CuPc) exhibits local enhancement of exciton emission in MoS_2_.^18^ Weiss and co-workers^19^ also reported that the photoinduced charge transfer between CuPc and MoS_2_ is strongly dependent on the face-on orientation of CuPc on the (1L)MoS_2_ surface. Moreover, the charge separation lasts about 17 times longer than the metal-free Pc/MoS_2_.

A very interesting phenomenon occurs on the interface between a (1L)MoS_2_ crystal and ZnPc: optically excited singlet exciton in ZnPc transfers to MoS_2_, forming a charge-transfer exciton, and singlet–triplet decay occurs due to the large singlet–triplet splitting in ZnPc and strong spin–orbit coupling in MoS_2_. The reported spin-selective back electron transfer may enable manipulation of the electron spin in hybrid electronic devices.^20^

Recently, the spin-dependent tunneling barriers, which can be manipulated through modifications of interface coupling, were proposed in mixed heterostructures composed of CoPc and a 2D magnet (VSe_2_).^21^

Surprisingly, the interaction of other technologically important classes of magnetic molecules and TMDs has not been addressed. If one takes into account the valley-selective response of (1L)TMDs, coupling of the valley-related pseudospin to another spin entity may result in prospective functionalities within a single hybrid material.

Under this proposition, we selected the terbium(III)bis(phthalocyaninato) (TbPc_2_) complex, which is one of the widely studied single molecule magnets (SMMs) with significant importance in the field of quantum information technologies. In standalone TbPc_2_, a Tb(III) ion is sandwiched between two Pc ligands, and the molecular spin dynamics and magnetization reversal are driven by an energy barrier produced by crystal field splitting of the *J* = 6 ground multiplet.^22^ TbPc_2_ shows strong magnetic anisotropy, with an easy axis of magnetization perpendicular to the Pc plane.^22^ However, the interaction of TbPc_2_ with its surroundings changes the magnetic response dramatically. For example, enhancement of the magnetic bistability of this SMM anchored to the silicon surface was observed,^23^ while interaction with magnetic substrates led to a significant reduction of magnetic hysteresis.^24−26^ Regarding the interaction of TbPc_2_ with nanomaterials possessing interesting electronic properties, TbPc_2_ heterostructures with carbon nanotubes^26,27^ and graphene^28,29^ have been explored, with a focus on the quantum regime of the SMM emerging at low temperatures.

In this study, we report a prototype of hybrid layered material, a valley–spin hybrid (VSH) system composed of TbPc_2_ and (1L)MoS_2_. We used comprehensive characterization techniques, including optical and photoelectron spectroscopies, to explore the mutual TbPc_2_/(1L)MoS_2_ interaction and the impact of the doping, strain, and defects on the helicity-resolved optical response of the TMD component bearing the valley contrast. We observed a strongly enhanced *A*-exciton-related valley polarization at room temperature, and we proposed a theoretical model to explain this mechanism. Thus, the mix-dimensional VSH materials show great opportunities thanks to their functionalities, such as chiral light emission revealed already at room temperature.

## Results and Discussion

### Preparation and Basic Characterization

**Figure 1:**
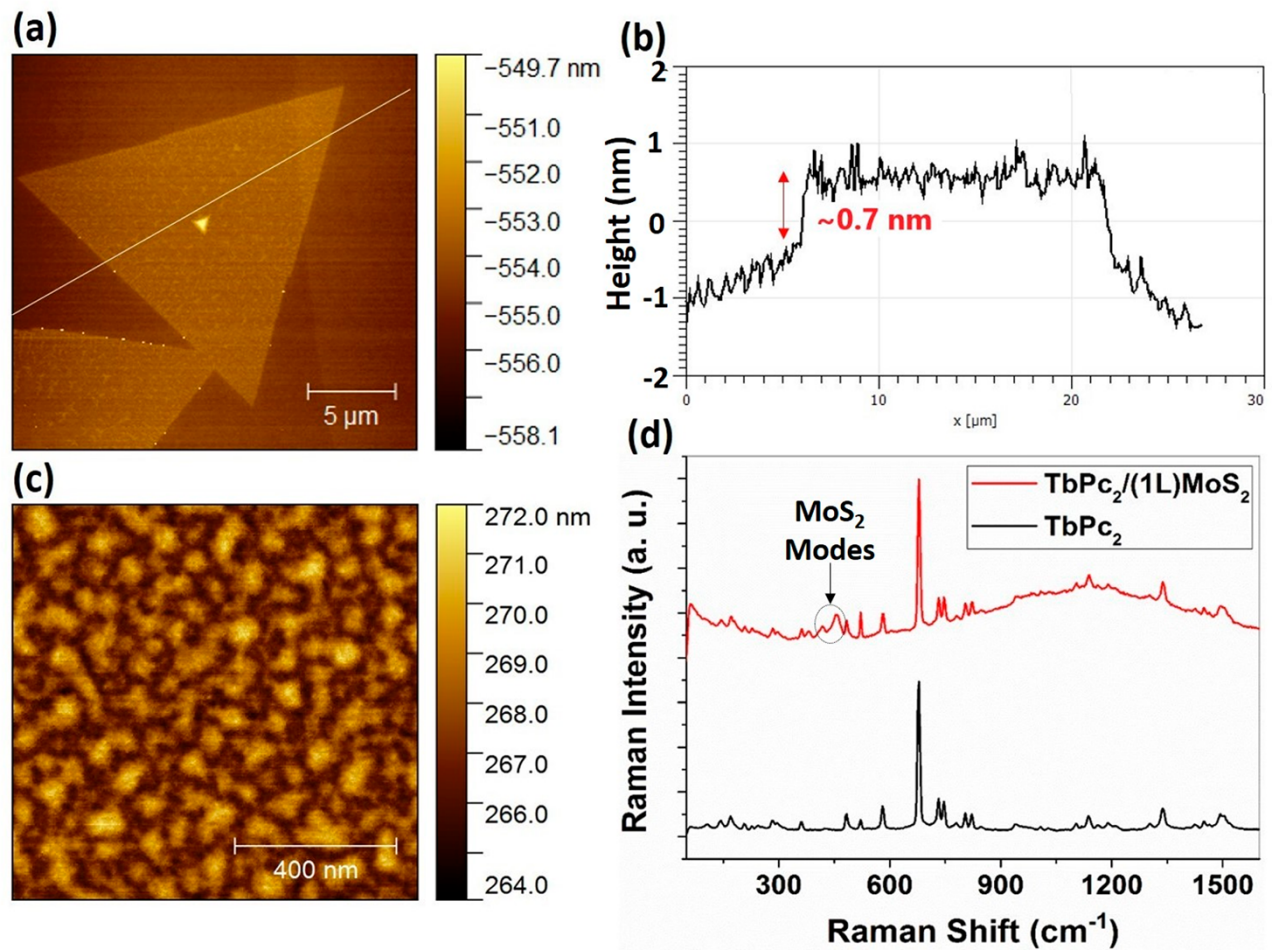
Results of topographic and spectroscopic characterization of (1L)MoS_2_ and TbPc_2_/(1L)MoS_2_. (a) A topographical AFM image of (1L)MoS_2_ demonstrating a triangular flake shape. The white line crossing the flake corresponds to the sample area depicted for the profile analysis. (b) Thickness profile of (1L)MoS_2_ taken along the line shown in panel a. (c) A topographic AFM image of the TbPc_2_ layer deposited on (1L)MoS_2_. (d) Respective Raman spectra of TbPc_2_ and TbPc_2_/(1L)MoS_2_ VSH (excitation laser energy of 2.33 eV).

First, the (1L)MoS_2_ flakes were synthesized by chemical vapor deposition (CVD; for more details, please see Section S3) and inspected by optical microscopy, atomic force microscopy (AFM), and Raman/PL microspectroscopy. Observation with the optical microscope revealed that the samples had large coverage by (1L)MoS_2_, and the grains in the continuous areas were found to be triangular in shape. The topographic AFM image of an isolated (1L)MoS_2_ flake is shown in Figure 1a, and the thickness profile along the (1L) flake is given in Figure 1b. The AFM data revealed that the thickness of the (1L)MoS_2_ sheet was about 0.7 nm, which is comparable to that reported in the literature.^30^

In the next step, the TbPc_2_ molecules were characterized by ultraviolet–visible (UV–vis) spectroscopy, Raman spectroscopy, and magnetic measurements (please see Section S1) and deposited onto (1L)MoS_2_ by thermal evaporation. About half of the sample area was covered before the deposition of TbPc_2_ in order to obtain a reference area of bare (1L)MoS_2_ and SiO_2_/Si substrate. Figure 1c shows the topography of the TbPc_2_ layer deposited by thermal vacuum evaporation onto (1L)MoS_2_. The root-mean-square (rms) roughness was found to be 0.74 nm.

The presence of TbPc_2_ on (1L)MoS_2_ after deposition was further confirmed by Raman microspectroscopy, carried out on the TbPc_2_ and TbPc_2_/(1L)MoS_2_ areas of the sample. The corresponding Raman spectra are presented in Figure 1d. The Raman peaks of the TbPc_2_ layer are located between 100 and 1600 cm^–1^ and can be attributed to the characteristic bending and stretching vibration modes of the TbPc_2_, in agreement with the literature (please see Table S1 for selected Raman active modes of TbPc_2_).^31^ For example, peaks located at 677, 730, 1136, and 1336 cm^–1^ correspond to Pc breathing, C–H wagging, pyrrole breathing, and pyrrole stretching, respectively. The Raman spectrum of TbPc_2_/(1L)MoS_2_ was found to be a superposition of individual spectra of TbPc_2_ and (1L)MoS_2_, displaying a fluorescence effect in higher wave numbers. The peaks corresponding to the in-plane *E′* and out-of-plane *A*_1_^′^ Raman modes of (1L)MoS_2_ were observed at 384 and 402 cm^–1^, respectively. The most striking differences in the Raman and PL spectra of (1L)MoS_2_ and TbPc_2_/(1L)MoS_2_ are discussed in more detail later in this section.

As a next step, X-ray photoelectron spectroscopy (XPS) measurements were used to investigate the chemical and electronic properties of TbPc_2_/(1L)MoS_2_. Figure S4a–c shows the core-level spectra for C 1s, N 1s, and Tb 3d_3/2_ of TbPc_2_/(1L)MoS_2_ and TbPc_2_ on Si/SiO_2_ substrate. A comparison of spectra from the samples prepared on a different piece of substrates shows negligible energy shifts and small shape variations. This indicates minor interactions between the central Tb atom and the environment. This finding is consistent with a previous study of similar double-decker systems.^32^ The Tb 3d_3/2_ peak’s binding energy at 1276.4 eV (Figure S4b) is also consistent with the previously reported value for TbPc_2_.^23,32,33^ The only discernible difference between the samples can be found in the shape of the N 1s spectra, which were fitted by two components. The low-energy (LE) component at 398.8 eV can be associated with “bulk-like” molecules not in direct contact with the surface, while the high-energy (HE) component at 400.2 eV can be associated with molecules affected by the substrate.^23,34^Figure S4c shows that, for the TbPc_2_/(1L)MoS_2_ sample, LE/HE intensity is lower than that for the TbPc_2_ sample. This finding is in agreement with the overall lower concentration of TbPc_2_ species on the surface of the TbPc_2_/(1L)MoS_2_ sample (please see the relative atomic concentration in Table S2). The photoelectron spectrum of Mo 3d recorded for the TbPc_2_/(1L)MoS_2_ sample (Figure S4d) did not reveal any significant change in comparison to the spectra usually obtained from CVD (1L)MoS_2_ samples. A small amount of molybdenum oxide detected in the spectra is a common residual from CVD growth.

The X-ray reflectivity (XRR) study of TbPc_2_/(1L)MoS_2_ (Figure S5) enabled us to determine the rms, which was found to be 0.56 nm (comparable to the results obtained by AFM). Analysis of the XRR curves yielded the electron density profile, which suggested that the molecules at the layer in the proximity of (1L)MoS_2_ tend to orient with the high-symmetry axis perpendicular to the (1L)MoS_2_ layer, as has been reported for TbPc_2_ on graphene,^28^ and the degree of order decreases with the distance from the substrate.

### Photoluminescence and Raman Microspectroscopy Investigations

**Figure 2:**
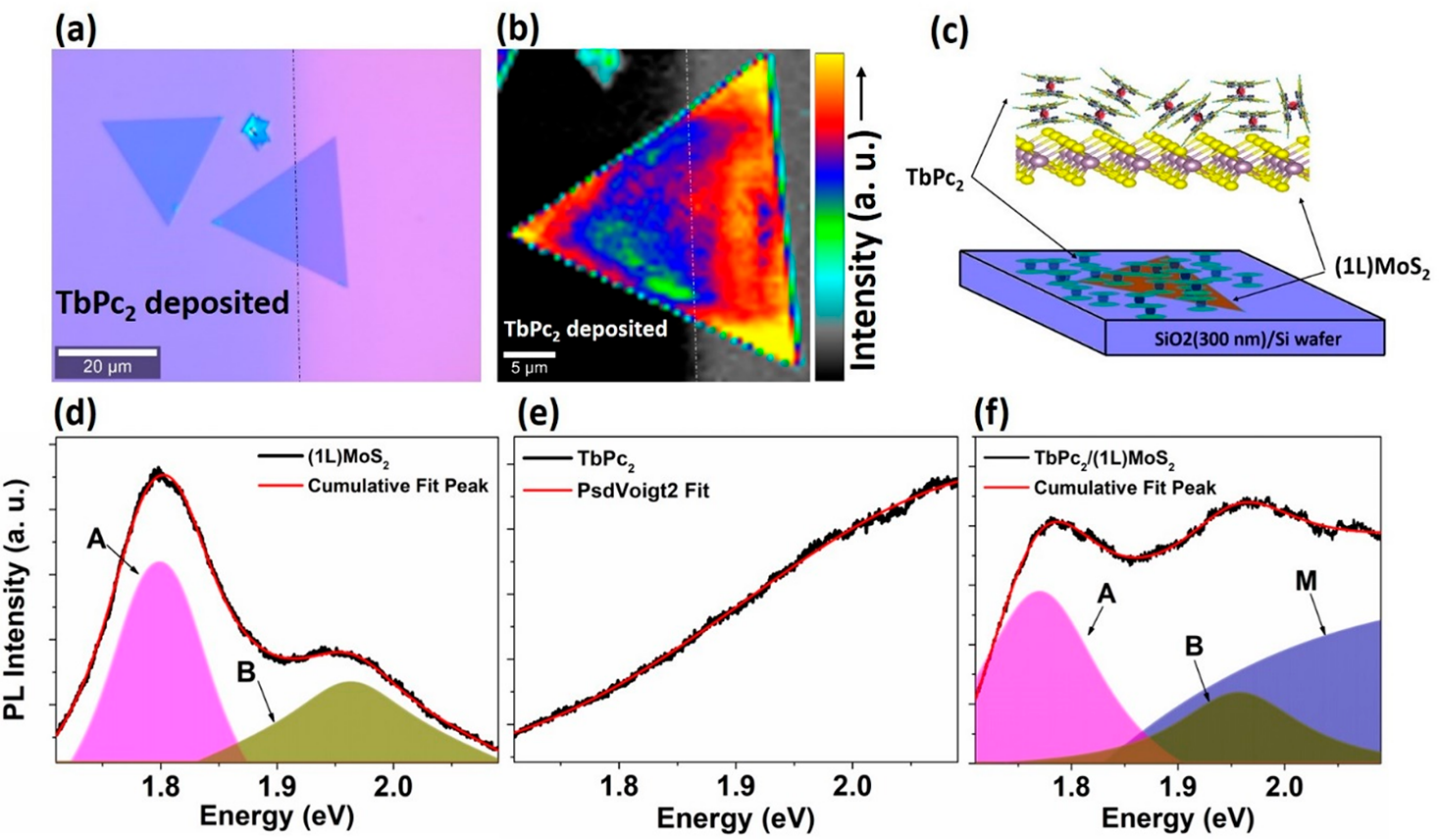
Optical and spectroscopic inspection of the TbPc_2_/(1L)MoS_2_ VSH in comparison to the reference areas. (a) The optical image of TbPc_2_ deposited on (1L)MoS_2_. (b) Average PL map depicting TbPc_2_ covered and noncovered areas on (1L)MoS_2_ single crystal, with the intensity scale bar. (c) A sketch of TbPc_2_/(1L)MoS_2_ on the SiO_2_(300 nm)/Si wafer. The bottom row shows PL spectra (d) for (1L)MoS_2_, (e) for TbPc_2_, and (f) for TbPc_2_/(1L)MoS_2_ with the fit using Pseudo–Voigt (PsdVoigt) profile functions, where *A*, *B*, and *M* denote neutral *A* exciton, *B* exciton, and contribution from TbPc_2_, respectively.

The successfully prepared TbPc_2_/(1L)MoS_2_ VSH was further investigated by room-temperature Raman and photoluminescence (PL) microspectroscopy. Figure 2a shows an optical image of a (1L)MoS_2_ flake in which approximately the left half of the single crystal is covered with TbPc_2_ molecules. This region on the edge of the molecular layer was selected to obtain a direct comparative analysis between VSH TbPc_2_/(1L)MoS_2_ and neat (1L)MoS_2_ within the same single-crystalline flake. The average PL intensity map plotted on the 610–710 nm scale is shown in Figure 2b. The color code clearly reveals that the TbPc_2_ molecules induce quenching in the PL spectra of (1L)MoS_2_. It can be seen that the average PL intensity is slightly lower in the left portion of the crystal covered with TbPc_2_ than in the unexposed area. This PL quenching may result from a possible charge transfer at the heterojunction of TbPc_2_/(1L)MoS_2_ before any exciton decay occurs. Similar PL quenching has been reported for other 2D materials decorated with metal phthalocyanines. The effect was rationalized by the value of the redox potential of the molecules, which was situated below the conduction band minima (CBM) of the 2D monolayer.^35^ Consequently, it can be suggested that the redox potential of TbPc_2_ lies below the CBM of (1L)MoS_2_ as well. As the PL intensity from the 2D crystal itself is usually nonuniform due to defects and the spatial distribution of doping and strain (as discussed further in the following sections), the quenching also slightly varies across the inspected area. The quenching phenomena can be explained by Q-band absorption of TbPc_2_; however, charge transfer can occur along with Q-band absorption in such heterostructures.

Representative PL characteristics for the (1L)MoS_2_, TbPc_2_, and TbPc_2_/(1L)MoS_2_ structures are shown in Figure 2d–f, respectively, along with deconvolution of the peaks and a cumulative fit to the overall spectra. The PL of (1L)MoS_2_ is characterized by a peak around 670 nm (1.82 eV), which is attributed to the *A* exciton (ground state exciton) and a shoulder peak around 630 nm (1.95 eV) resulting from the *B* exciton (coming from the higher energy spin–orbit split state). The ratio of the *A* and *B* exciton peak intensities can be considered as a tentative measure of the quality of the single crystalline flake.^12,36^ The low intensity of the *B* exciton peak suggests that (1L)MoS_2_ has a relatively small number of defects. The PL spectra from the TbPc_2_ molecular layer on the SiO_2_/Si substrate presented in Figure 2e show a gradual increase in intensity toward lower wavelengths, which can be attributed to excitons resulting from the highest-occupied/lowest-unoccupied molecular orbital (HOMO/LUMO) levels of the TbPc_2_ molecules.

Finally, Figure 2f displays the PL spectrum of the VSH heterostructure TbPc_2_/(1L)MoS_2_ on the SiO_2_/Si substrate along with its deconvolution. The intensity of the defect peak (i.e., *B* exciton) is elevated due to superposition of the excitons from the molecular layer. Although the PL spectrum of the VSH seems to be roughly a superposition of the individual spectra of (1L)MoS_2_ and TbPc_2_, more detailed analysis revealed that the *A* exciton peak is moderately red-shifted by 3–5 nm in TbPc_2_/(1L)MoS_2_ in comparison to bare (1L)MoS_2_, which suggests a weak interaction between the (1L)MoS_2_ and TbPc_2_ molecules.

To investigate the impact of the TbPc_2_ molecules on the *A* and *B* excitons individually, PL maps of about 30 μm × 30 μm on different 2D single crystals of (1L)MoS_2_ with and without TbPc_2_ were studied (Figure S6). Typically, the PL spectra of (1L)MoS_2_ were dominated by *A* excitons. The PL map of average intensity over the *A* exciton peak in (1L)MoS_2_ compared to that of TbPc_2_/(1L)MoS_2_ with the same scale is presented in Figure S6a,b, where overall quenching can be observed.

Interestingly, the average intensity of the *B* exciton peak appears to show a reverse effect, where the presence of TbPc_2_ molecules on (1L)MoS_2_ leads to enhancement of the *B* exciton intensity, as can be seen in Figure S6c,d, respectively. However, this effect can be explained by the contribution of the PL from TbPc_2_, as there is only a tiny change in the *B* exciton peak due to TbPc_2_ deposition, as illustrated previously. Figure S6e,f compares the average peak position of the *A* exciton as a function of energy for (1L)MoS_2_ and TbPc_2_/(1L)MoS_2_, respectively. The red-shift in TbPc_2_/(1L)MoS_2_ can be observed clearly. The reduction in the PL photon energy of the *A* exciton peak in TbPc_2_/(1L)MoS_2_ (∼0.04 eV) suggests a moderate optical band gap change, substantiating the p-doping effect of TbPc_2_ molecules on (1L)MoS_2_, which is corroborated by the Raman spectra analysis discussed below.

Raman spectroscopy is arguably the most common technique for analyzing doping, strain, electron density, and electron–phonon interactions in 2D materials. Typically, charge transfer, doping, and strain phenomena are correlated with the position and width of *E′* and *A*_1_^′^peaks in (1L)MoS_2_.^37^ The most prominent peaks in the Raman spectra of (1L)MoS_2_ are located at 384 and 402 cm^–1^ due to in-plane *E′* and out-of-plane *A*_1_^′^modes, respectively^18^ (Raman spectra extended to the 2LA mode area with negligible changes are shown in Figure S7).

**Figure 3:**
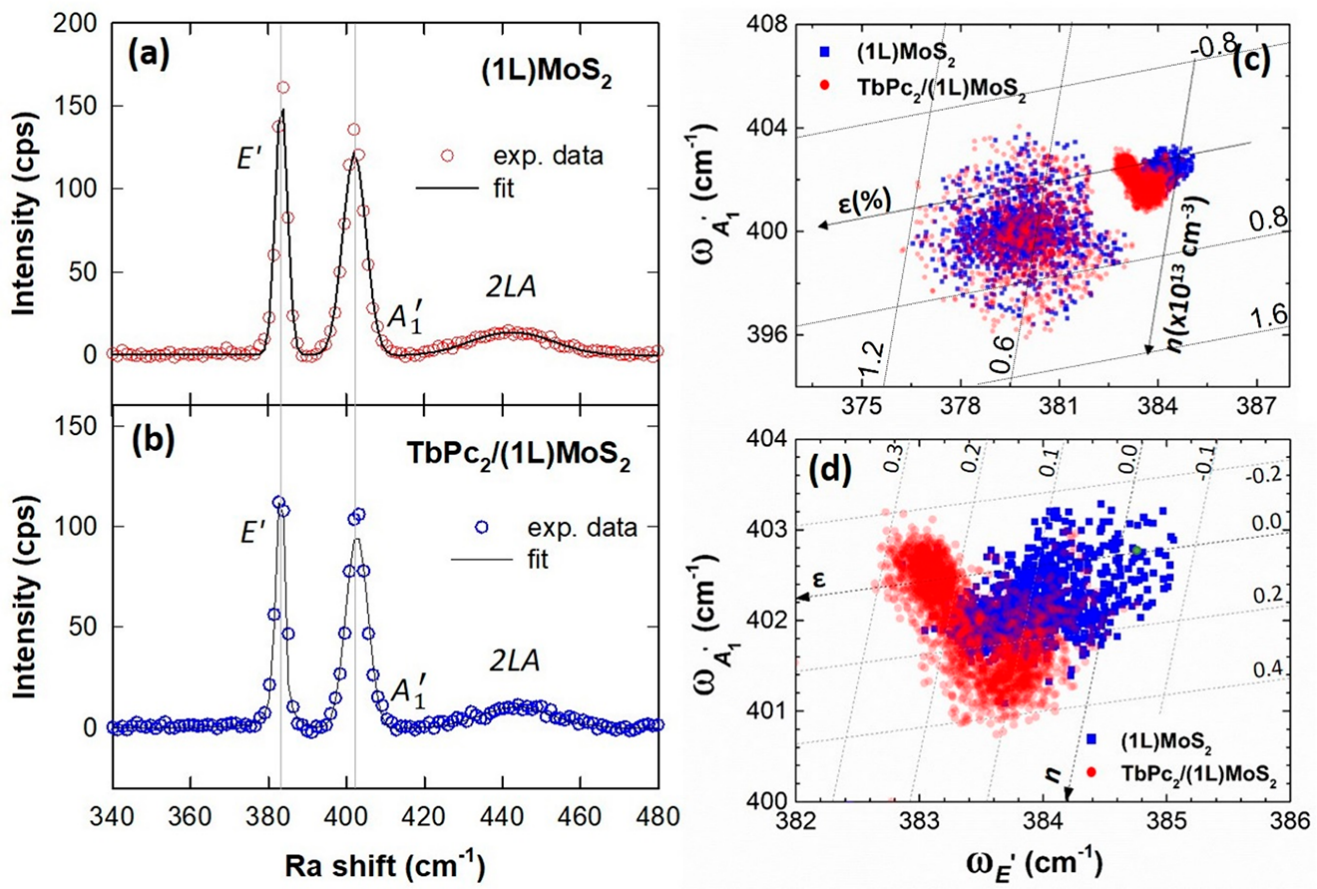
Results of Raman microspectroscopy investigations of TbPc_2_/(1L)MoS_2_. Typical Raman spectra together with the fitted curves shown (a) for (1L)MoS_2_ and (b) for TbPc_2_/(1L)MoS_2_. (c) Raman correlation plots: ω_*E*^′^_ vs ω_*A*_1_^′^_ for (1L)MoS_2_ with and without TbPc_2_ molecules are shown in the panel. (d) Enlarged portion of the correlation plot (c), showing the axis for doping level (*n*) and strain (ε), is shown in the panel. The *n* and ε are estimated as ×10^13^ cm^–2^ and percentages, respectively.

Figure 3a,b shows the comparison between the Raman spectra of pristine (1L)MoS_2_ and TbPc_2_/(1L)MoS_2_. The *A*_1_^′^peak reveals a blue-shift by 3–4 cm^–1^ in TbPc_2_/(1L)MoS_2_ in comparison to the pristine (1L)MoS_2_, which is in agreement with the expected p-doping of (1L)MoS_2_. Interestingly, the *E′* peak was marginally red-shifted by 1–2 cm^–1^, which has been explained as competition between the van der Waals force and dielectric shielding in van der Waals (vdW) heterostructures.^38^

In order to obtain more insight into the influence of TbPc_2_ molecules on the (1L)MoS_2_, a Raman correlation analysis was performed for pristine (1L)MoS_2_ and TbPc_2_/(1L)MoS_2_ (Figure S8 shows the Raman shift distribution over the flakes for the *E′* and *A*_1_^′^peaks). To extract the information related to the strain and doping effects, a correlation diagram of the Raman shifts of the *E′* and *A*_1_^′^modes, which are both influenced by doping and strain, was constructed (Figure 3c,d). The data points for (1L)MoS_2_ and TbPc_2_/(1L)MoS_2_ are plotted in blue and red, respectively.

There are two major lobes present in the correlation diagram for each sample area. The first symmetrical lobe around *E′* ∼ 380 cm^–1^ and *A*_1_^′^ ∼ 400 cm^–1^ is almost identical for both (1L)MoS_2_ and TbPc_2_/(1L)MoS_2_. This more diffusive contribution mostly arises from the edges of the flakes. The second lobe is asymmetric; its zoomed version is shown in Figure 3d along with the superimposed iso-strain, ε and iso-doping, and *n* lines, where the green dot represents the reference for the Raman shift of the pristine-like (mechanically exfoliated) (1L)MoS_2_.^39^ The defined axis of strain and doping level have been defined previously using Grüneisen parameters of different Raman modes.^39,40^ Closer inspection of Figure 3d suggests that the data points of the top right lobe can be further divided into two sublobes with a different slope. The top left lobe has a major contribution from the central region of the flake, while the bottom right lobe corresponds to the edges of the triangular flake. The doping *n* is moderately increased due to the presence of TbPc_2_ on (1L)MoS_2_, and the values of *n* are ∼0.1 × 10^13^ cm^–1^, indicating p-type doping. There is also a slight increase in the strain of about 0.1%, which is more pronounced around the central region of the flake.

To underpin the charge carrier dynamics in (1L)MoS_2_ and TbPc_2_/(1L)MoS_2_, time-resolved PL (TRPL) measurements were performed using the fluorescence lifetime imaging (FLIM) technique. The TRPL observations (Figure S11) also corroborate the presence of the photoinduced charge-transfer phenomena discussed above. The appearance of an additional slow decay channel in TbPc_2_/(1L)MoS_2_ is associated with the intersystem charge transfer, which introduces an additional delay, and this is a commonly observed phenomenon in layered heterostructures.^41−43^ For more details, please see Section S2.5.

### Valley-Selective Emission under CP Light

**Figure 4:**
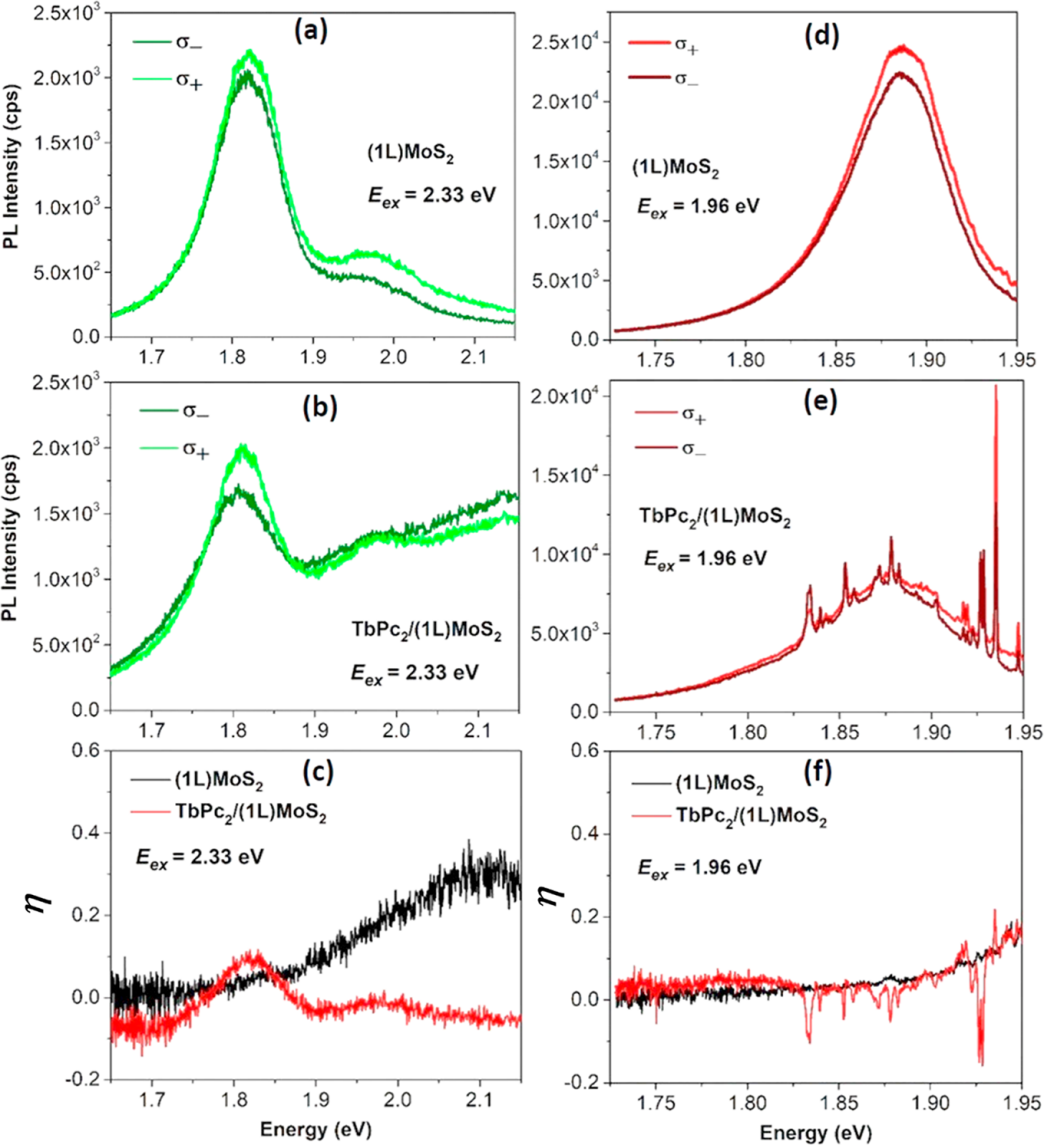
Valley polarization analysis of (1L)MoS_2_ and TbPc_2_/(1L)MoS_2_: (a, b) show the excitation of (1L)MoS_2_ and TbPc_2_/(1L)MoS_2_, respectively, in off-resonance at *E*_*ex*_ = 2.33 eV. (c) The valley polarization (η) as a function of energy estimated from the respective intensities at *E*_*ex*_ = 2.33 eV. (d,, e) Show the excitation of (1L)MoS_2_ and TbPc_2_/(1L)MoS_2_, respectively, in off-resonance at *E*_*ex*_ = 1.96 eV. (f) The valley polarization, η, as a function of energy at *E*_*ex*_ = 1.96 eV.

One of the most striking properties of the TMD monolayers is the strong valley contrast associated with the emission of circularly polarized photons. In order to explore the influence of TbPc_2_ on the valley-selective emission of (1L)MoS_2_, we recorded polarization-resolved PL spectra of as-grown (1L)MoS_2_ and TbPc_2_/(1L)MoS_2_ using CP light. We would like to point out that, despite the significant magneto-optical properties of TbPc_2_, the degree of polarization is not affected by these effects for the given experimental geometry and stacking of the molecules in the layer. The broken spin degeneracy along with time-reversal symmetry define the spin and valleys in (1L)MoS_2_, which can be optically excited with the helicity of CP light (left-handed (σ^–^) and right-handed (σ^+^)) in a selective manner.^44,45^ In the first experiment, the PL spectra were recorded under the σ^+^ CP laser excitation of energy, *E*_*ex*_ = 2.33 eV (off-resonant condition) (see Figure 4a,b). The emission energy dependence of the degree of polarization has been determined as η = (*I*(σ^+^) – *I*(σ^–^))/(*I*(σ^+^) + *I*(σ^–^)), where *I*(σ^+^, σ^–^) are the polarization-resolved PL intensities.

The calculated η dependencies are shown in Figure 4c for the off-resonant case. A significantly lower degree of polarization of the *A* and *B* excitons was observed in the as-grown (1L)MoS_2_. This is not surprising, as the degree of valley polarization η in (1L)MoS_2_ is expected to be negligible at room temperature and in the out-of-resonance condition.^45−47^ On the other hand, in the TbPc_2_/(1L)MoS_2_ heterostructure, η was found to be strongly enhanced. This observation is in contrast to the previously observed CP emission from (1L)MoS_2_ under off-resonant excitation. Thus, the η enhancement cannot be explained by the intrinsic valley and spin selective response of (1L)MoS_2_ to the CP light, and the observed effect must come from the light–matter interaction with the VSH.

We further performed a polarization-resolved experiment under the pumping of close-to-resonance (*E*_*ex*_ = 1.96 eV) with *B* exciton (∼1.98 eV). The results are shown in Figure 4d,e for (1L)MoS_2_ and TbPc_2_/(1L)MoS_2_, respectively. The corresponding degree of polarization, η, has been shown in Figure 4f. Please note that the PL spectra for the VSH structure are superimposed by the Raman bands of the TbPc_2_ molecules. A different result, yet consistent with previously observed ones, is obtained: the polarization gradually increases up to ∼0.18 in both cases, apart from the “negative Raman glitches” from the TbPc_2_ molecules in the VSH structure. This observation is consistent with the previous observation of off-resonant excitation due to the fact that the close-to-resonant excitation produces significantly fewer photocarriers in the TbPc_2_ excited state. Thus, the polarization dependence of (1L)MoS_2_ remains independent of TbPc_2_. The effect persists down to low temperatures and under external magnetic fields (see Figure S10).

### Mechanism for Valley-Polarization Enhancement

Prior to entering a deeper discussion of the observed phenomenon, let us briefly summarize the important features of the band structure and related optical transitions of the TbPc_2_ molecule.^48^ In TbPc_2_, about 2.6 electrons are transferred from the metal to the Pc ligands. The charge transferred from the Tb ion to the ligands is not sufficient to saturate them, as each Pc plane requires two electrons to be saturated. The corresponding unpaired electron has π-character and is delocalized over both ligands. Consequently, the degenerated state occupied by a π-radical is split into a singly occupied molecular orbital (SOMO) state below and LUMO state above the Fermi level with opposite spin indices due to the Pauli Exclusion Principle.^48,49^ The TbPc_2_ reveals two strong π → π* optical transitions from the SOMO to the LUMO+1 (Q_1_) and HOMO to the LUMO (Q_2_) orbitals with the absorption maxima located at 1.86 and 2.00 eV, respectively, giving rise to the well-known Q-band.^50^

Moreover, the decoupling between the Tb and ligand spin systems is responsible for the conservation of the electronic properties of the molecule deposited on various substrates. Therefore, we can expect that the main features of the electronic band structure will not be drastically modified due to the interaction with (1L)MoS_2_.^48,49,51^

**Figure 5:**
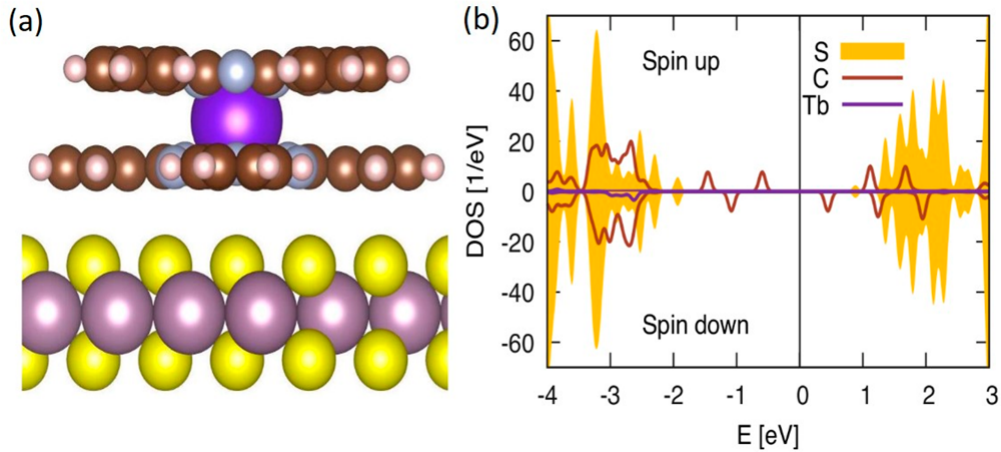
(a) Ball-and-stick model of the relaxed TbPc_2_/MoS_2_ structure (S = yellow, Mo = violet, C = brown, H = pink, N = gray, and Tb = magenta). (b) Density of states projected on the S, C, and Tb atoms for both spin directions from the density functional theory (DFT) calculation. The zero energy separates the occupied and unoccupied states. An artificial Gaussian broadening of the resonances is applied.

To elucidate the nature of the chemical bond and low-lying excitations of the (1L)MoS_2_/TbPc_2_ interface, we resort to density functional calculations using a hybrid functional PBE0.^52^Figure 5a shows the relaxed geometry, where the Tb atom sits on top of the sulfur. The relaxed distance between the lowest Pc and topmost S layers is ∼3.3 Å, typical for physisorption. The Pc planes remain parallel as a result of the vdW attraction.

Figure 5b also shows the density of states (DOS) projected on S, C, and Tb atoms. The S contribution to the DOS indicates the edges of the valence and conduction bands. We see that the gap size does not change due to adsorption (up to few percent) by comparing to the DOS of pristine MoS_2_. The DOS of the molecular species is devoid of any Tb contribution within the bulk gap. The DOS of C shows four in-gap peaks occupied by three electrons, i.e., carrying spin-half moment. This is the ligand spin, known from TbPc_2_ in isolation. Thus, the overall charge transfer to the molecule is moderate and orbital unspecific. Consequently, we conclude that the electronic structure of the adsorbed molecule is only slightly perturbed compared to the molecule in isolation. Thus, the major mechanism behind the chiral light emission is originated from the valley-related excitons in (1L)MoS_2_.

In order to understand the inequity in the optical intensities for the emission upon the irradiation with the circularly polarized light, we use the pseudospin formalism to explore the dynamics of the bright exciton doublet in the MoS_2_/TbPc_2_ heterostructure. The approach considers competing relaxation mechanics from exciton exchange and from single-particle spin flip into optically inactive states. In this approach, the 2 × 2 density matrix ρ_**q**_ of MoS_2_ excitons with center-of-mass momentum **q** = (*q* cos ϕ, *q* sin ϕ, 0) is presented in the form:
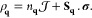
1Here, *n*_**q**_ = Tr[ρ_**q**_]/2, the pseudospin-average-distribution function of the excitons,  is the 2 × 2 unity matrix, (**S**_**q**_)_*j*_ = Tr[ρ_**q**_σ_*j*_]/2, where *j* = *x*, *y*, and *z* are components of the pseudospin, σ_*j*_ is the Pauli matrix, and “.” is the state for the scalar product. The *S*_*z*_ component describes the valley polarization, while the *S*_*x*_ and *S*_*y*_ components define the valley coherences of the excitons in the monolayer. The pseudospins satisfy the Maialle-Silva-Sham (SMM) equation:^53^
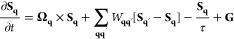
2The term **Ω**_**q**_ = (Ω_∥_ cos(2φ), Ω_∥_ sin(2φ),0) represents the effective pseudospin precession frequency. It is originated from the long-range exchange interaction between the exciton states of different valleys (see details in ref (54)). *W*_**qq′**_ is the scattering rate induced by the impurities, exciton–phonon interaction, and exciton–exciton interaction, τ represents the decay time of the excitons, and **G** describes the external source of the valley coherence (*G*_*x*_, *G*_*y*_) and valley polarization (*G*_*z*_) in the system.“×” stands for the vector product. Solving the MSS equation for *G*_*z*_ ≠ 0, *G*_*x*_ = *G*_*y*_ = 0, one can observe that the steady-state valley polarization η ∝ *S*_*z*_ is suppressed by Ω even in the absence of the scattering *W*_**qq′**_ = 0:
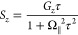
3This expression also demonstrates the decreasing of the valley coherence with the larger decay time τ. Since the parameter τ contains the contribution from the radiative decay as well as from nonradiative processes, its value can be dependent on the details of the experiment and the studied samples.

For example, the effective exciton decay time in TMDs can depend on temperature.^55^ Namely, at the low-temperature limit, the thermalized excitons are localized at the radiative zone *q* < *q*_rad_ = ω_ex_/*c* according to the Boltzmann distribution. Here, ω_ex_ is the minimal frequency of light, which causes bright exciton transitions. Such excitons mainly decay radiatively with τ_rad_ of a few picoseconds. At high temperatures, the situation is changed, because a part of the exciton population is localized outside of the radiative region and forms the nonradiative reservoir. To depopulate this reservoir, the excitons from nonradiative states must be relaxed to the radiative region. Such a relaxation process includes the exciton–phonon interaction and takes much longer time τ_eff_, up to nanoseconds.^56^ This time can be effectively incorporated in the MSS equation by replacing the second and third terms in eq 2 by – **S**_**q**_/τ_eff_. Therefore, for this case, one can estimate the strong decrease of the valley polarization by replacing τ with τ_eff_ in eq 3.

In the experiments with the (1L)MoS_2_/TbPc_2_ heterostructure, the parameters Ω_**q**_ and *W*_**qq′**_ cannot depend on the frequency of the incident light. Hence, we conclude that the observed difference in valley polarizations of (1L)MoS_2_/TbPc_2_ can appear only from the effective relaxation times.

Let us consider two experimental cases and analyze the possible modification of the relaxation times in each case. In the first case, the heterostructure is shined upon by the light of the energy *E* = 2.33 eV. This energy is much larger than the energy of the Q_1_ (*E*_Q1_ = 1.86 eV) and Q_2_ (*E*_Q2_ = 2 eV) transitions in the TbPc_2_ molecule. Hence, this component remains unexcited, and all the light energy is transferred to the monolayer and generates the so-called hot excitons. These excitons lose their energy due to exciton–phonon and other many-body interactions and reach, in particular, the lowest energies of *A* (*E*_A_ = 1.78 eV) and *B* (*E*_B_ = 1.98 eV) transitions in the studied heterostructure. Both transitions are clearly visible.

Moreover, the *A* exciton line demonstrates the nonzero valley polarization η ≈ 20%. The experiment is done at room temperature *T* = 300 K, and one can assume that phonons of MoS_2_ are in thermodynamic equilibrium. However, these phonons cannot affect the phonons in TbPc_2_, since the vibrational modes of both layers are not coupled, as confirmed by Raman spectroscopy (see Figure 1d). Hence, the molecules cannot be excited and equilibrated by the phonons of MoS_2_. The only way to excite the molecular layer is the Coulomb-induced near-resonant energy transfer between excitons of the MoS_2_ and TbPc_2_ layers. This special process modifies the dynamics of the excitons in the considered system.

**Figure 6:**
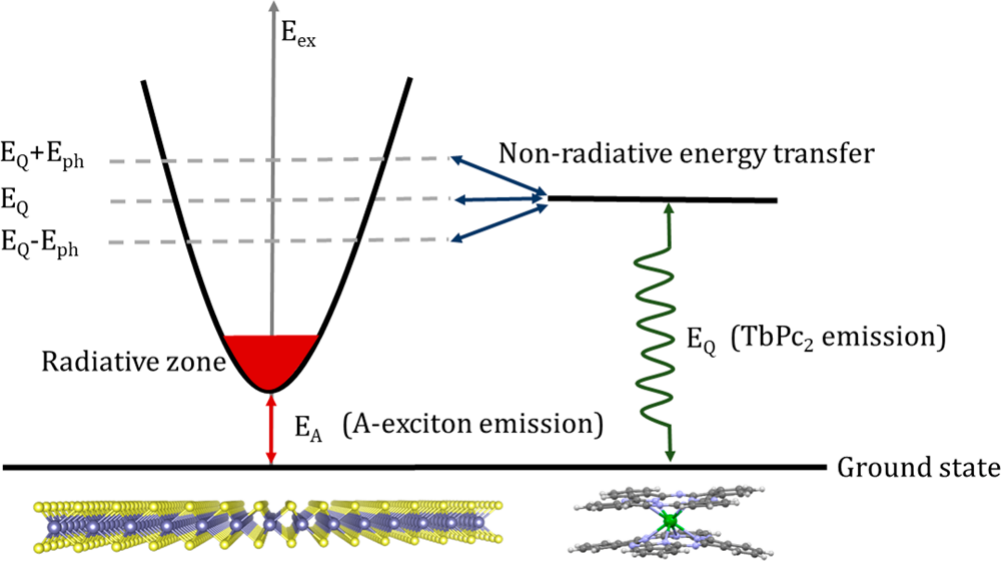
Schematic illustration of the nonradiative energy drain mechanism. The scheme shows the nonradiative energy transfer between the TMD component (left) and TbPc_2_ molecular magnet (right) together with the essential radiative optical processes in both components of the VSH.

Considering that excitons can emit or absorb phonons with average energy *E*_ph_(*T*) at temperature *T*, we conclude that excitons in the energy domain Δ*E* ∈ [*E*_Q1_ – *E*_ph_(*T*), *E*_Q1_ + *E*_ph_(*T*)] nonradiatively decay by transferring their energy to the molecules. The average phonon energy can be estimated as *E*_ph_(*T*) ≈ *k*_B_*T* = 26 meV. Since *E*_Q1_ – *E*_ph_(*T*) – *E*_A_ ≈ 54 meV ≫ (*ℏq*_rad_)^2^/2*m*_ex_ ≈ 3 μeV (*m*_ex_ is the exciton mass), the excitons in the radiative region are not affected by such process. In other words, the molecular layer works as an “energy drain” for the nonradiative excitonic states giving rise to a mechanism schematically visualized in Figure 6.

The drained energy excites the TbPc_2_ molecules, which re-emit photons of broad energy range which, however, does not overlap with the energy of the *A* exciton. Therefore, these photons do not give a contribution to the valley polarization of the *A* exciton. The TRPL analysis presented in Figure S11 supports these findings. This emission explains naturally the similarity of the TRPL decay profiles for MoS_2_/TbPc_2_ and TbPc_2_ layers, presented in Figure S11e,f, respectively. Since the reservoir of nonradiative excitons is depleted by the energy drain process, none of them scatter into the radiative region and increase the effective lifetime of the excitons in the radiative zone. It results to the nonzero valley polarization in the *A* exciton emission. A similar effect of long-lived exciton filtering was considered earlier for the case of the graphene/TMD heterostructures.^57^

Finally, similar arguments cannot be applied to the *B* excitons, since *E*_Q2_ – *E*_B_ = 20 meV is comparable with the phonon energy *E*_ph_ ≈ 26 meV. In this case, the energy transfer between the radiative zone of the *B* excitons and TbPc_2_ molecules is possible and the dynamics of the non- and radiative excitons remain coupled.

In the second type of experiment, the studied heterostructure is shined by the light of energy *E* = 1.96 eV. This energy is close to the energy of the Q_2_ transition, and the molecular layer can be excited in this case. Due to relaxation processes in the magnetic molecules, the lowest energy Q_1_ type transitions are also activated, leaving no room for energy transfer from the excitons of the monolayer. Therefore, the aforementioned energy drain is blocked in such a case, and the excitons experience dynamics as in the regular monolayer at temperature *T* = 300 K. Such dynamics leads to near-zero valley polarization η ≈ 0 at *T* = 300 K due to a large effective exciton decay time.

## Conclusions and Outlook

In conclusion, we propose a class of layered hybrid materials composed of 2D semiconductors and single-molecule magnets with intrinsic diverse spin (valley) degrees of freedom. The prototype VSH was successfully prepared by thermal evaporation of TbPc_2_ molecules on (1L)MoS_2_ grown by a one-step CVD process on the SiO_2_/Si substrate. The persistence of the TbPc_2_ molecules after deposition was unambiguously confirmed using both microscopic and spectroscopic techniques (AFM, Raman and PL microspectroscopy, XRR, and XPS). The interaction of TbPc_2_ and (1L)MoS_2_ spin components was explored using Raman and PL microspectroscopy. We clearly observed that the PL signal is moderately quenched in the VSH compared to the pristine (1L)MoS_2_, which is attributed to a superficial charge transfer between TbPc_2_ and (1L)MoS_2_. Correspondingly, a moderate red-shift observed for the *A* exciton implies an optical bandgap in TbPc_2_/(1L)MoS_2_ changes. TRPL and Raman spectroscopy results also point to the presence of p-type doping due to photogenerated electron transfer between the TbPc_2_ molecule and (1L)MoS_2_.

Nevertheless, the most striking observation is that the interaction of the TbPc_2_ molecules with (1L)MoS_2_ enhances the polarization of the *A* exciton-related valley, despite an off-resonance condition and room temperature. To explain this unanticipated effect, we propose a nonradiative energy drain mechanism in which the population of the nonradiative excitons is depleted via interaction with the unoccupied electronic states of the molecular magnet. This process partly blocks the scattering of the nonradiative excitons in the radiative region and does not increase the effective lifetime of the excitons in the radiative zone, giving rise to the nonzero valley polarization in the *A* exciton-related emission.

Thanks to the fascinating properties, feasible production, and great stability, the layered hybrid materials composed of monolayer TMDs and double-decker molecule magnets represent multiresponsive and multioperable opto-spin-valletronic platforms capable of chiral light emission, exploitable in the emerging proposals for information storage and quantum computing.

## Methods

### Growth of (1L)MoS_2_

(1L)MoS_2_ was synthesized on SiO_2_/Si substrate using the one-step CVD method in a horizontal single-zone furnace at atmospheric pressure. Details of this process and the experimental setup are provided in Section S3. The morphology and size of the (1L)MoS_2_ monolayers were characterized by an optical microscope. The thickness of (1L)MoS_2_ was found to be ∼0.7 nm using AFM profilometry.

### Preparation of TbPc_2_

The [TbPc_2_]^0^ complex was synthesized according to the published procedure.^58^ A mixture of 1,2-dicyanobenzene (15.6 mmol), Tb(acac)_3_·4H_2_O (1.9 mmol), and DBU (7.8 mmol) in 20 mL of pentan-1-ol was refluxed for 2 days. The solution was allowed to cool down to room temperature; 1.5 mL of acetic acid was added, and the mixture was heated at 100 °C for 0.5 h. After cooling, the precipitate was collected by filtration and washed with *n*-hexane and Et_2_O. The crude product was dissolved in 300 mL of the CHCl_3_/MeOH mixture (1:1, v/v), and PcH_2_ was filtered off as a purple solid. The reaction mixture was then left overnight, adsorbed on active basic alumina oxide, and purified by column chromatography on basic alumina oxide deactivated with 4.6% of H_2_O (level IV) with CHCl_3_/MeOH (10:1, v/v) as an eluent. After an additional purification by column chromatography on silica gel (CHCl_3_/MeOH, 10:1, v/v), the [TbPc_2_]^0^ complex, as a dark green solid, was precipitated from the CHCl_3_/*n*-hexane mixture, filtered off, and dried in a vacuum. The neutral state of the complex was confirmed by UV–vis spectroscopy (presented in Figure S1). The pristine powder of TbPc_2_ was also characterized with Raman spectroscopy (Figure S2) and magnetic measurements (Figure S3).

### Preparation of TbPc_2_/(1L)MoS_2_ VSH

The SiO_2_/Si substrate with (1L)MoS_2_ was taken to an organic evaporator, and a thin layer with a nominal thickness of ∼9 nm of TbPc_2_ was subsequently evaporated at 400 °C onto the (1L)MoS_2_ at ∼10^–6^ mbar of pressure to obtain a TbPc_2_/(1L)MoS_2_ heterostructure. A reference sample of TbPc_2_ on SiO_2_/Si substrate was obtained using the same protocol. The ∼9 nm thickness of the TbPc_2_ thin film and the orientational order of the molecules in the TbPc_2_ layer were further confirmed with XRR. The presence of TbPc_2_ was also confirmed by XPS and Raman spectroscopy.

### Characterization Methods

AFM images and thickness profiles of the structures were acquired using Bruker’s AFM Dimension ICON system in the quantitative nonmechanical mode. A Bruker silicon tip was used to acquire AFM data, and Gwyddion software was used to process and analyze the AFM images.

In the XRR experiment, specular X-ray reflection was measured on a rotating-anode X-ray diffractometer RIGAKU Smartlab (45 kV, 200 mA) using Cu Kα radiation and a parabolic multilayer mirror on the primary side; the angular resolution of the whole experimental setup was 0.005°. The reflection curves were fitted to a three-layer model. For each layer, we determined its thickness (*T*_n_), root-mean square (rms) roughness σ_n_, and the electron density ρ_n_ relative to the fixed electron density of the Si substrate, ρ_Si_. For the fitting procedure, we used standard reflectivity software based on the Nevot-Croce and Parrat formulas.^59^

Magnetization measurements were performed with a Physical Property Measurement System equipped with a vibrating sample magnetometer (VSM) (Quantum Design) in the temperature range of 2–300 K with an applied magnetic field up to 14 T. The powder sample (mass ∼ 2 mg) was placed in an original Quantum Design sample holder for VSM and pressed carefully to minimize movement of the powder particles during the experiments.

For UV–vis characterization, a UV–vis-NIR PerkinElmer Lambda 1050 spectrometer was used. The samples were dissolved in chloroform and measured in quartz cuvettes.

The XPS measurements were performed using a VG ESCA3MkII electron spectrometer with a base pressure over 10^–9^ mbar. Al Kα radiation was used for the excitation of the electrons. The electrons were energy analyzed using a hemispherical analyzer operating at a constant pass energy of 20 eV. The estimated error in binding energy determination was ±0.1 eV. The spectra were calibrated by setting the C 1s peak belonging to hydrocarbon and/or adventitious carbon at the binding energy of 285 eV. The Mo 3d, C 1s, N 1s, and Tb 3d_3/2_ photoelectrons were measured. The Tb 3d_3/2_ line is the only one usable to confirm the presence of Tb due to the overlap of the Tb 4d line with Si 2s and the overlap of the 3d_5/2_ line with the KVV Auger peak of carbon. The spectra were curve-fitted using the Gaussian–Lorentzian line shape after subtraction of the Shirley background. The surface atomic content was accomplished assuming a homogeneous distribution of atoms, Wagner sensitivity factors, and corrections for the analyzer transmission function.

### Optical Microspectroscopies

Raman and PL spectra of both pristine (1L)MoS_2_ and TbPc_2_/(1L)MoS_2_ were collected in the backscattering geometry with an excitation wavelength of 532 nm (2.33 eV) at 200 μW power using a WITec spectrometer at room temperature. Flakes with a scale size of about 30 μm were selected and mapped for PL and Raman spectra with a spatial resolution of 0.5 μm using a grating of 600 and 1800 lines/mm, respectively.

CP-PL and Raman measurements were carried out in LabRAM HR Evolution (Horiba) by exciting the samples with σ^+^ helicity. The CP Raman spectra for TbPc_2_/(1L)MoS_2_ were obtained using an incident excitation of 532 nm (2.33 eV), while CP-PL was carried out with energy excitations of both 633 nm (1.96 eV) and 532 nm (2.33 eV). A λ/4 plate was placed just before the objective, and the incoming orientation was chosen using a λ/2 plate, whereas the outgoing orientation was selected using a polarizer. The helicity of the light impacting the sample was optimized using a Thorlabs TXP 5004 polarimeter, whereas the helicity of the scattered light was optimized by searching for the minimum (or maximum) intensity of the Rayleigh line reflected from a thick, sputtered Au film. It is well-known that the performance of a charge coupled device (CCD) is strongly dependent on the helicity of the incoming light. In order to avoid these polarization sensitivities of the CCD, we put a depolarizer in front of the detector. The CP-PL and CP Raman spectra were obtained using 150 and 1800 lines/mm gratings, respectively.

The temperature-dependent Raman and PL spectra were measured in the backscattering geometry using a low-temperature confocal Raman microscope insert (attoRAMAN, attocube) placed in a Physical Property Measurement System (PPMS, Quantum Design). A low temperature and magnetic field compatible 100× objective (numerical aperture 0.82 and lateral resolution of 500 nm) lens were used to focus the 632.8 nm HeNe laser beam of pump fluence ∼ 250 μW. The low-frequency Raman spectra were obtained down to ±10 cm^–1^ by combining two volume Bragg grating notch filters into the Raman system to suppress the Rayleigh signal. The incident laser beam was circularly polarized using a set of standard 633 nm half and quarter-wave plates. Similarly, a series of broadband quarter and half-wave plates have been used to obtain polarization-resolved emitted signals. The degree of polarization was precalibrated for each incident and scattered beam (ellipticity > 44.5°) using ThorLabs’s TXP polarimeter. The spectral resolution of the spectrometer under the measurement condition using 1800 lines/cm grating was 0.6 cm^–1^. The intensity response of the CCD detector was precalibrated using a tungsten halogen light source (HL-2000-CAL, Ocean Optics). The spectra integration times were 120 s to achieve good signals. Apart from the PPMS temperature readout, the actual temperature of the sample was monitored through a thermometer mounted on the optical insert at the sample position, and the spectra were recorded while the temperature stabilized at the measured point during the cooling cycle from 3000 to 10K K.

TRPL measurements were recorded with an Olympus FluoView1000 confocal system coupled to a PMT detector (tau-SPAD, Picoquant) with subnanosecond time correlated single photon counting (TCSPC) capability (HydraHarp 400, Picoquant). A long-pass filter (edge at 580 nm) was used to record the lifetimes of both the structures. The samples were excited with a 532 nm laser (2.33 eV; LDH-P-FA Series, Picoquant) with a power of 6.5 nW μm^2^, a frequency of 40 MHz, and a pulse duration of 80 ps. Consequently, FLIM was also performed, and the lifetime maps were acquired. The decay profiles were fitted with a single-exponential decay function.

### DFT Calculations

Kohn–Sham states were represented using a localized basis set, as implemented in the FHI-AIMS package.^60^ Relativistic corrections were included at the scalar level in the atomic zero order regular approximation.^60^ To account for exchange correlation, we employed the PBE functional^61^ for the geometry relaxation and PBE0 functional^52^ for calculating the density of states. We checked our results against the B3LYP functional^62^ and did not find qualitative changes. All calculations included the *C*_6_/*R*^6^ van der Waals contribution to the forces from the Tkatchenko–Scheffler partitioning.^63^ We used the BFGS algorithm for relaxation, until the absolute value of the maximum force component dropped below 0.01 eV/Å. We started the structural relaxations with the molecule situated in diverse lateral positions (on-S, on-Mo, hollow) and diverse angles relative to the crystalline directions. The result was always a physisorption, as depicted in the main text. We estimated the charge transfer to the molecule using the Mulliken technique, yielding less than 2% electrons.

## Supporting Information Available

Additional results related to the synthesis and characterization of TbPc_2_, MoS_2_, and TbPc_2_/(1L)MoS_2_; optical and magnetic characterization of TbPc_2_; XPS and X-ray reflectivity data of TbPc_2_/(1L)MoS_2_ along with additional Raman/PL/valley polarization (with *E*_*X*_ = 2.33 eV) analysis and time-resolved PL data; CVD growth of MoS_2_ (PDF)
