# Supplementary material for: Chiral Light Emission from a Hybrid Magnetic Molecule–Monolayer Transition Metal Dichalcogenide Heterostructure
Source: ACS Nano. 2023 Jan 18;17(3):2170–81. doi: 10.1021/acsnano.2c08320 (PMC10017025; doi:10.1021/acsnano.2c08320)
Supplement: Supplementary file 1 — nn2c08320_si_001.pdf [file nn2c08320_si_001.pdf]

Supplementary Information

Chiral Light Emission from a Hybrid Magnetic Molecule–Monolayer Transition Metal Dichalcogenide Heterostructure

*Vaibhav Varade,[1] Golam Haider,[2] Artur Slobodeniuk,[1] Richard Korytar,[1] Tomas Novotny,[1] Vaclav Holy,[1] Jiri Miksatko,[2] Jan Plsek,[2] Jan Sykora,[2] Miriam Basova,[1] Martin Zacek,[1] Martin Hof,[2] Martin Kalbac,[2,#] Jana Vejpravova[1,]**

[1]Department of Condensed Matter Physics, Faculty of Mathematics and Physics,

Charles University, Ke Karlovu 5, 121 16, Prague 2, Czech Republic

*Email: jana@mag.mff.cuni.cz

[2]J. Heyrovsky Institute of Physical Chemistry, Dolejskova 3, 182 23, Prague 8, Czech Republic

[#]Email: martin.kalbac@jh-inst.cas.cz

S1. Additional characterization of $TbPc_2$

S1.1 UV-Vis characterization of $TbPc_2$

UV-Vis spectroscopy: The ultraviolet–visible (UV-Vis) spectrum of $TbPc_2$ recorded in dimethyl sulfoxide is shown in Figure S3. The most intense Q band at ~675 nm occurs due to transition from a semi-occupied molecular orbital to the second lowest-unoccupied molecular orbital (LUMO), and from the second fully occupied highest-occupied molecular orbital (HOMO) to the first LUMO. The electron dipole movement allows the $\pi \rightarrow \pi^*$ transition between the third occupied HOMO and the first LUMO giving rise to Soret band near blue wavelength region of the spectrum at around 338 nm. These signals indicate the neutral form of the complex: $[TbPc_2]^0$.

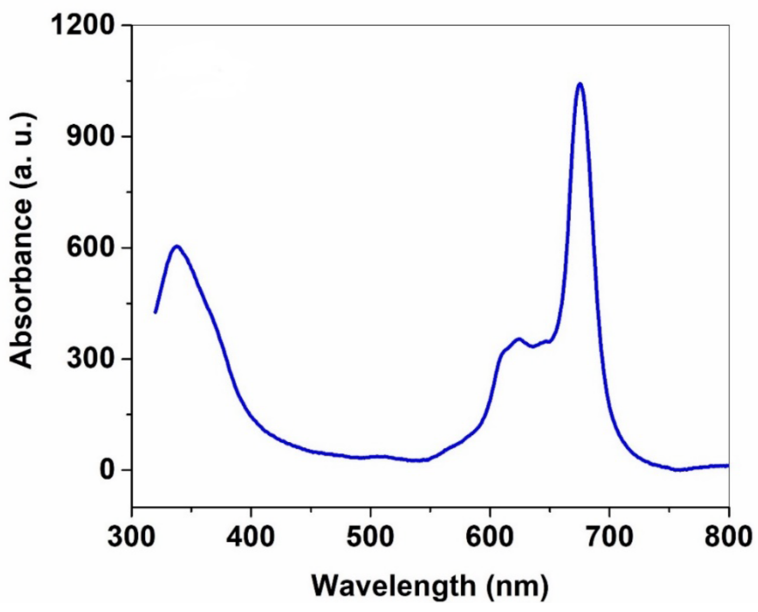

Figure S1. UV-Vis absorption spectrum of $TbPc_2$

S1.2 Raman spectroscopy characterization of $TbPc_2$

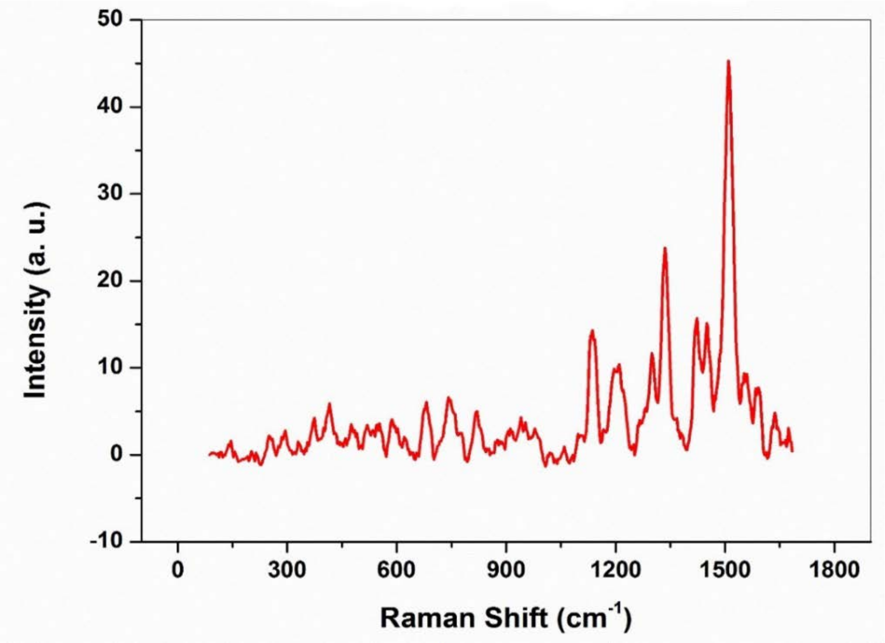

Figure S2. Raman spectrum of the $TbPc_2$ powder using 633 nm excitation

Table S1: Vibrational fundamental frequencies of $TbPc_2$

Raman modes (cm^{-1})	Interpretation
678	Pc breathing
740	C-H wag
778	Pc ring
1139	Pyrrole breathing
1300	C-H bending
1340	Pyrrole stretch
1448	Isoindole stretch
1520	C-N aza group

S1.3 Magnetic measurements of $TbPc_2$

Magnetic properties: Magnetization isotherm of $TbPc_2$ powder was measured in both polarities of magnetic field up to 14 T, the saturation value reaches about 4 μ_B as expected. Temperature-dependence of magnetic susceptibility as a function of temperature is shown in Figure S4. The fit by Curie-Weiss law at the high-temperature limit for J = 6 (Tb^{3+}) is also shown.

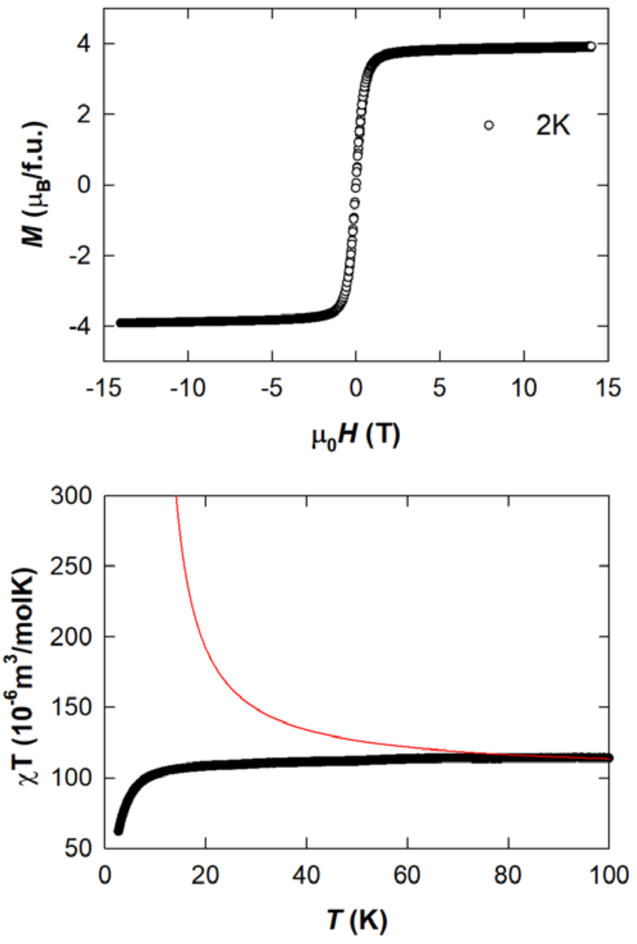

Figure S3. Magnetization isotherm recorded at 2 K (upper) and temperature dependence of magnetic susceptibility in χ representation (lower) of $TbPc_2$ molecule measured at 1 T (black points). Fit of the Curie-Weiss law (applied to the temperature interval, where the $1/\chi$ vs. T is linear) is shown by the solid red line.

S2. Additional characterization of spin hybrid

S2.1 XPS measurements

Results of XPS study carried out on a spin hybrid and a reference sample without (1L)MoS_2/$TbPc_2$ film (thickness 9 nm) on SiO_2/Si substrate). The XPS spectra are shown in Figure S5 and the estimation of the atomic concentration is given in Table S2.

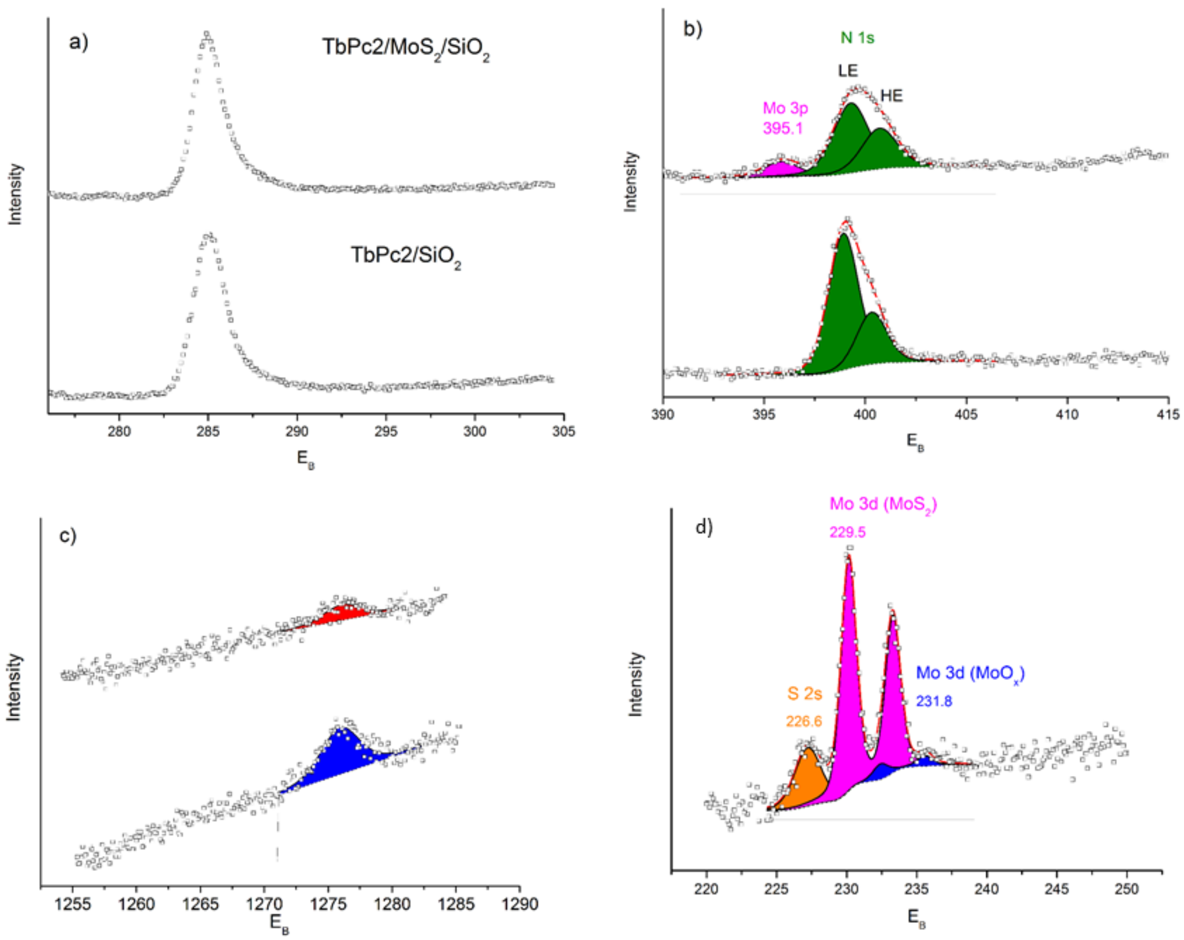

Figure S4. XPS spectra of the $TbPc_2$/(1L)MoS_2/SiO_2 and $TbPc_2$/SiO_2 samples. Panels (a-c) correspond to the C1s (a), N1s (b), and Tb $3d_{3/2}$ (c) XPS spectra of the $TbPc_2$/(1L)MoS_2/SiO_2. Mo 3d and S 2s XPS spectra of the $TbPc_2$/(1L)MoS_2/SiO_2 are shown in panel (d).

Table S2: Atomic concentration of Tb, N, C, Si, Mo and S elements estimated by XPS. First line corresponds to a reference sample composed of $TbPc_2$ film (thickness 9 nm) on SiO_2/Si substrate. The second row corresponds to the spin hybrid $TbPc_2$/(1L)MoS_2 on SiO_2/Si substrate.

Sample	Tb	N	C	Si	Mo	S
$TbPc_2$/SiO_2	0.31	13.40	83.33	2.96	-	-
$TbPc_2$/(1L)MoS_2/SiO_2	0.12	9.97	75.30	12.42	0.76	1.44

S2.2 X-ray reflectivity measurements

Results of XRR investigation of the spin hybrid are shown in Figure S6. The reflection curve was fitted to a model of two or three layers, for each layer we determined its thickness T, root-mean square (rms) roughness, σ and the electron density, ρ relatively to the fixed electron density of the Si substrate.

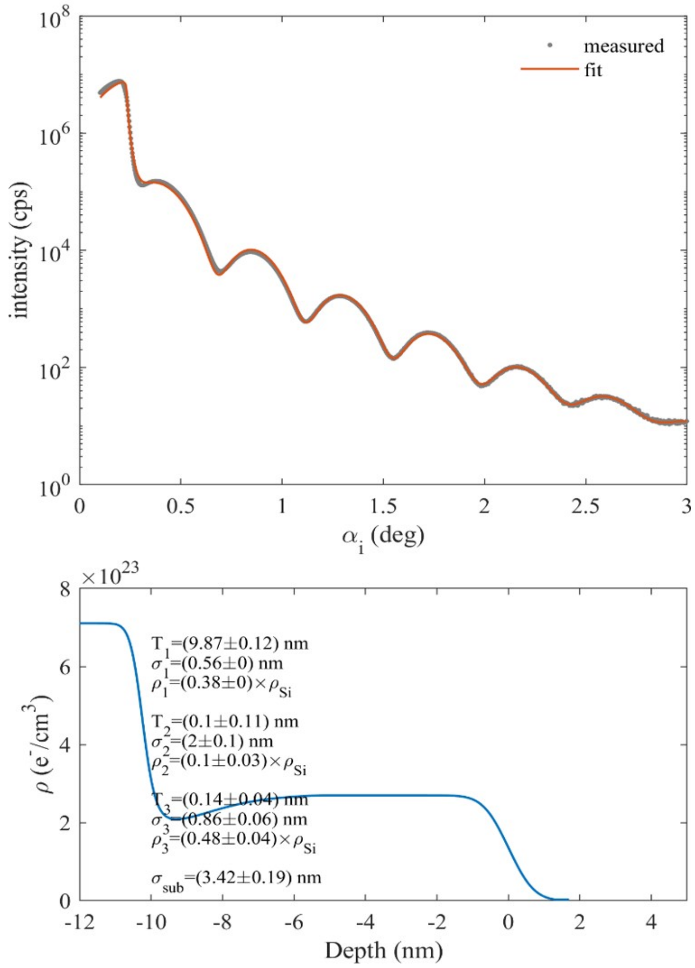

Figure S5. XRR data recorded on $TbPc_2$/(1L)MoS_2. The top panel corresponds to a reflectivity curve, while the bottom panel shows the electron density profile across the sample. Thickness (T), roughness, σ and relative density (ρ, scaled to Si density) is given in the panel for the $TbPc_2$ (index 1), (1L)MoS_2 (index 2) and SiO_2 (index sub). The indexes (n) refer to individual layers, n = 1 means the topmost layer at the sample surface, which is the $TbPc_2$.

S2.3 Conventional PL and Raman micro-spectroscopy

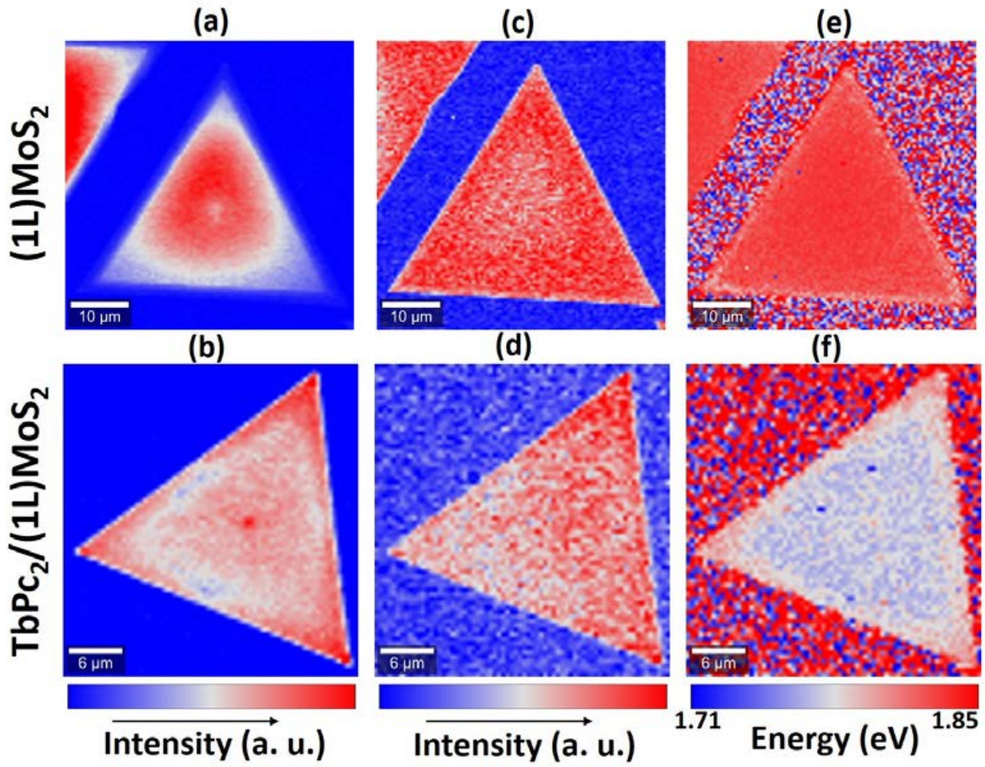

Figure S6. Results of PL micro-spectroscopy study carried out on the $TbPc_2$/(1L)MoS_2 spin hybrid at the area of full coverage of (1L)MoS_2 by $TbPc_2$ and at the area of bare (1L)MoS_2. A PL map of the average *A* exciton intensity depicted (a) for (1L)MoS_2 and (b) for $TbPc_2$/(1L)MoS_2. A PL map of the average *B* exciton intensity presented (c) for (1L)MoS_2 and (d) for $TbPc_2$/(1L)MoS_2. A PL map of the maxima peak position of the *A* exciton is shown (e) for (1L)MoS_2 and f) for $TbPc_2$/(1L)MoS_2.

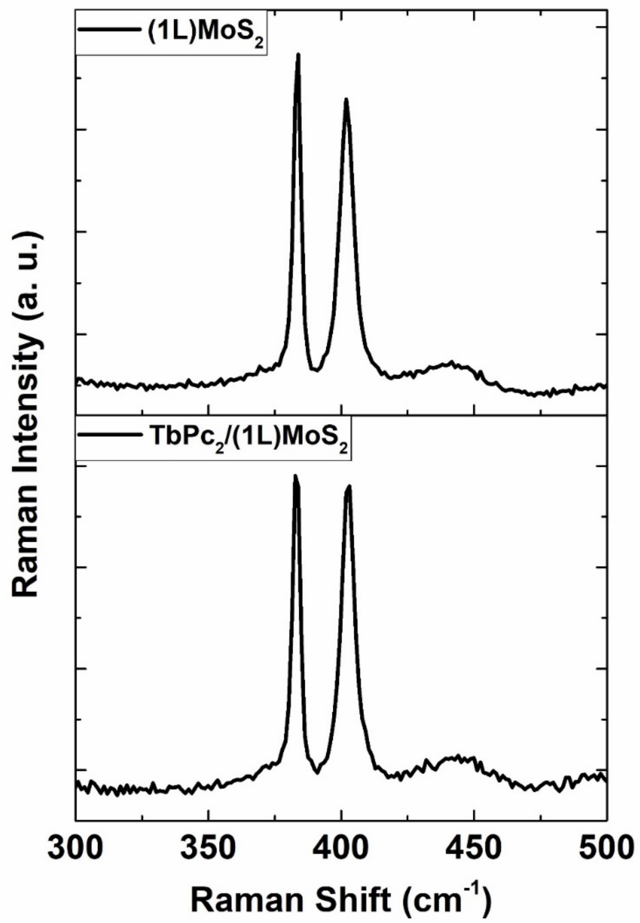

Figure S7. 2LA modes in the Raman spectra of the MoS_2 monolayer and the spin hybrid.

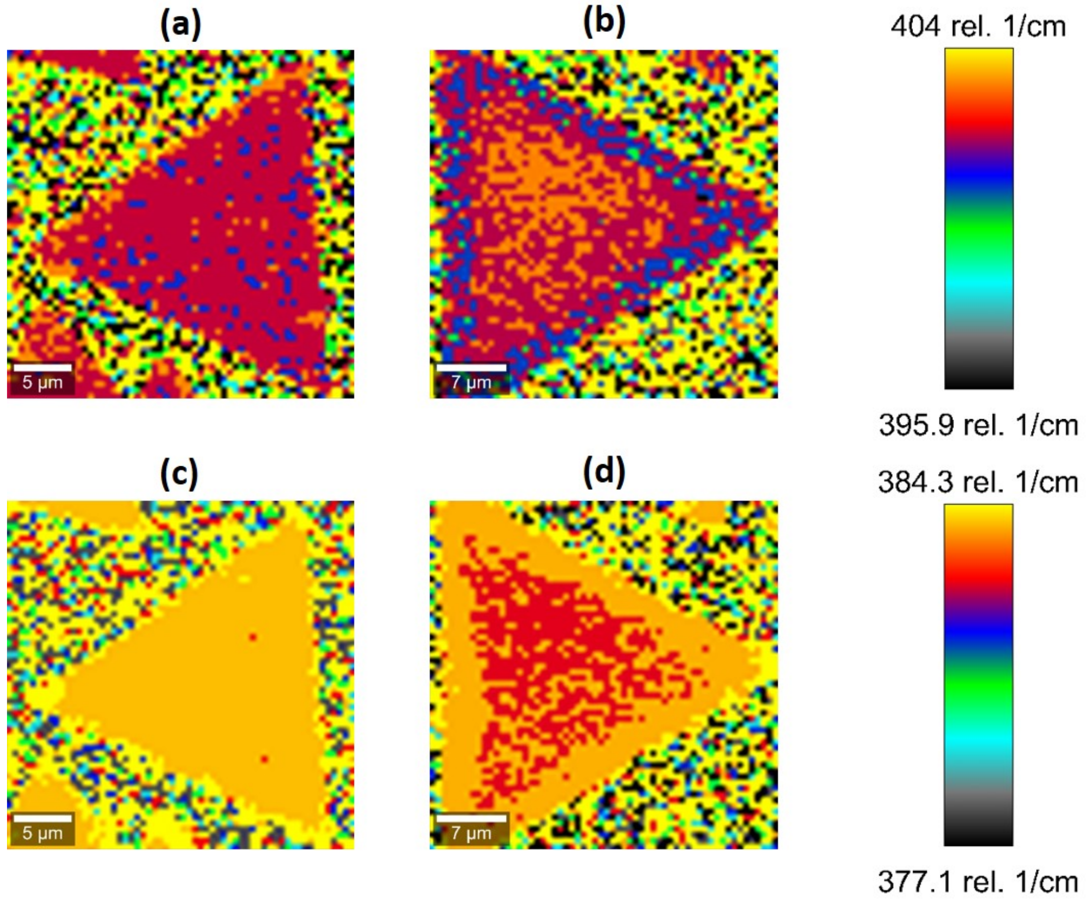

Figure S8. Raman spectral maps of the sample in the (1L)MoS_2 and $TbPc_2$/(1L)MoS_2 regions. (a) and (b) shows the distribution of the Raman shift around the A_1' peak for (1L)MoS_2 and $TbPc_2$/(1L)MoS_2, respectively. (c) and (d) shows the distribution of Raman shift about E' for (1L)MoS_2 and $TbPc_2$/(1L)MoS_2, respectively.

S2.4 Valley polarization analysis based on PL with CP light

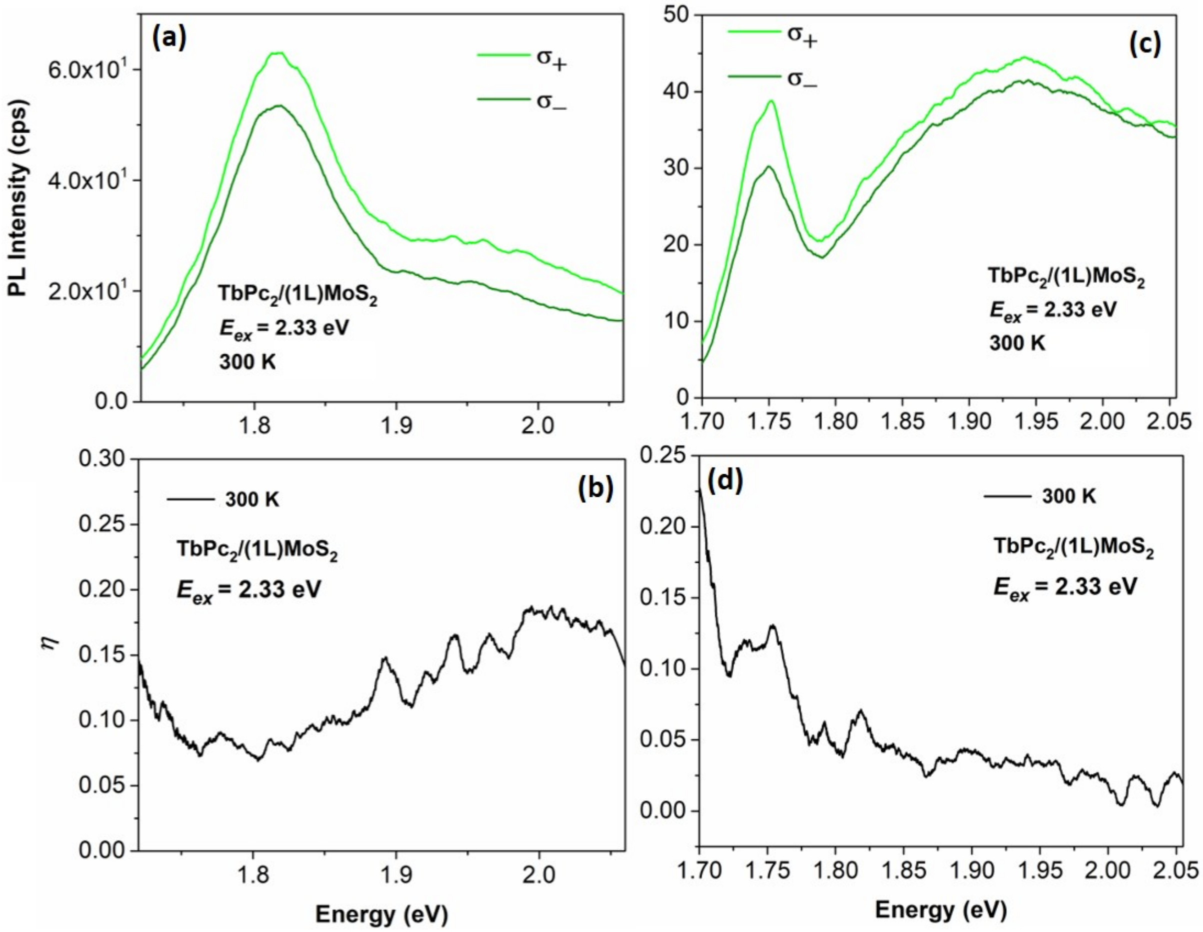

Figure S9. Valley polarization analysis of the $TbPc_2/(1L)MoS_2$ in off-resonance at E_{ex}=2.33 eV. (a), (c) - CP PL spectra and (b), (d) - valley polarization (η) obtained at different positions on the sample.

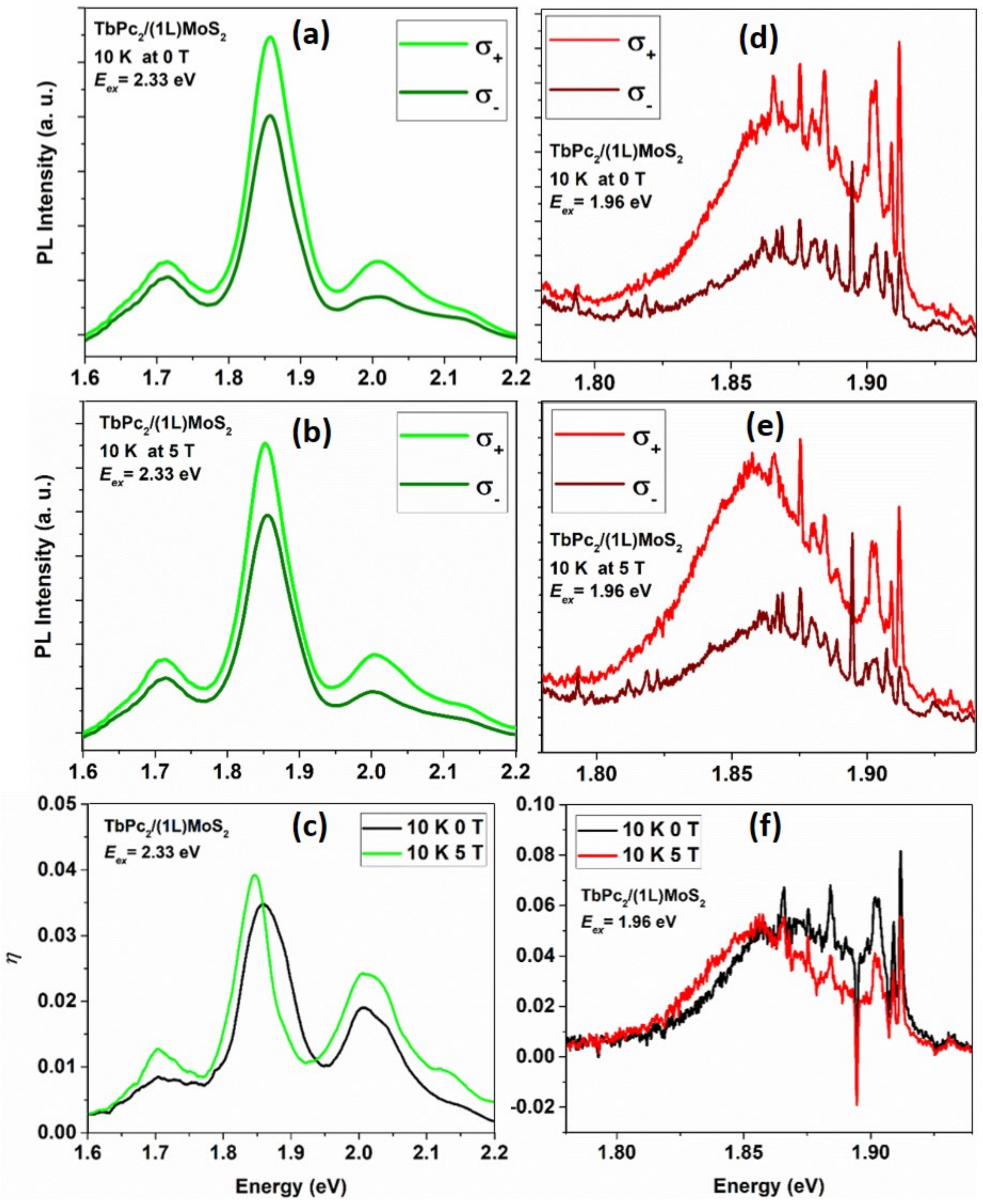

Figure S10. Valley polarization analysis of the (1L)MoS_2 and $TbPc_2$/(1L)MoS_2 at low temperatures (10 K): (a) and (b) show the excitation of (1L)MoS_2 and $TbPc_2$/(1L)MoS_2, respectively, in off-resonance at E_{ex}=2.33 eV. (c) The valley polarization (η) as a function of energy estimated from the respective intensities at E_{ex}=2.33 eV at 0 T and 5 T. (d) and (e) show the excitation of (1L)MoS_2 and $TbPc_2$/(1L)MoS_2, respectively, in off-resonance at E_{ex}=1.96 eV. (f) The valley polarization, η as a function of energy at E_{ex}=1.96 eV at 0 T and 5 T.

S2.5 Time-resolved PL

Figure S11a shows the transient PL decay dynamics while Figure S11b–S11c show spatially resolved fluorescence lifetime images of (1L)MoS_2, $TbPc_2$ and $TbPc_2$/(1L)MoS_2. The average excited state carrier lifetime was obtained by fitting a normal distribution function to the histograms in Figure S11d–S11f for (1L)MoS_2, $TbPc_2$ and $TbPc_2$/(1L)MoS_2, respectively. The lifetime spatial distribution on the (1L)MoS_2 flake shows that the edges have a slightly longer lifetime than the central region of the flakes, which is also reflected in the lifetime histogram as an asymmetric normal distribution. In (1L)MoS_2, the lifetime is estimated to be 0.62±0.10 ns at the center of the flake and 0.79±0.10 ns at the edge region. A similar trend is also observed in $TbPc_2$/(1L)MoS_2 spin hybrid (see Figure S11e), where the average lifetime is 0.69±0.02 ns at the center of the flake and 0.73±0.04 ns at the edges.

Moreover, the lifetime mapping shows that the deposition of $TbPc_2$ mainly affects the edges and center of the flake, which are usually more defective. Therefore, the $TbPc_2$ molecules tend to couple more strongly to the defects, which is in agreement with the Raman analysis, where the doping effects are more pronounced at the edges while strain is usually developed around the central region.[12]

Finally, a homogeneous lifetime distribution is observed in the region outside the (1L)MoS_2 flakes (Figure S11c), except for the locally enhanced values due to the few randomly distributed $TbPc_2$ agglomerates around the (1L)MoS_2 flakes on the substrate. The corresponding histogram presented in Figure S11f provides an average lifetime of 0.64±0.02 ns for the $TbPc_2$ film and 0.70±0.04 ns for the agglomerates on the substrates. The obtained lifetimes of 0.62±0.10 ns and 0.64±0.02 ns in (1L)MoS_2 and $TbPc_2$, respectively, are due to the overall radiative decay channels, which are consistent with the previously reported values.[1]

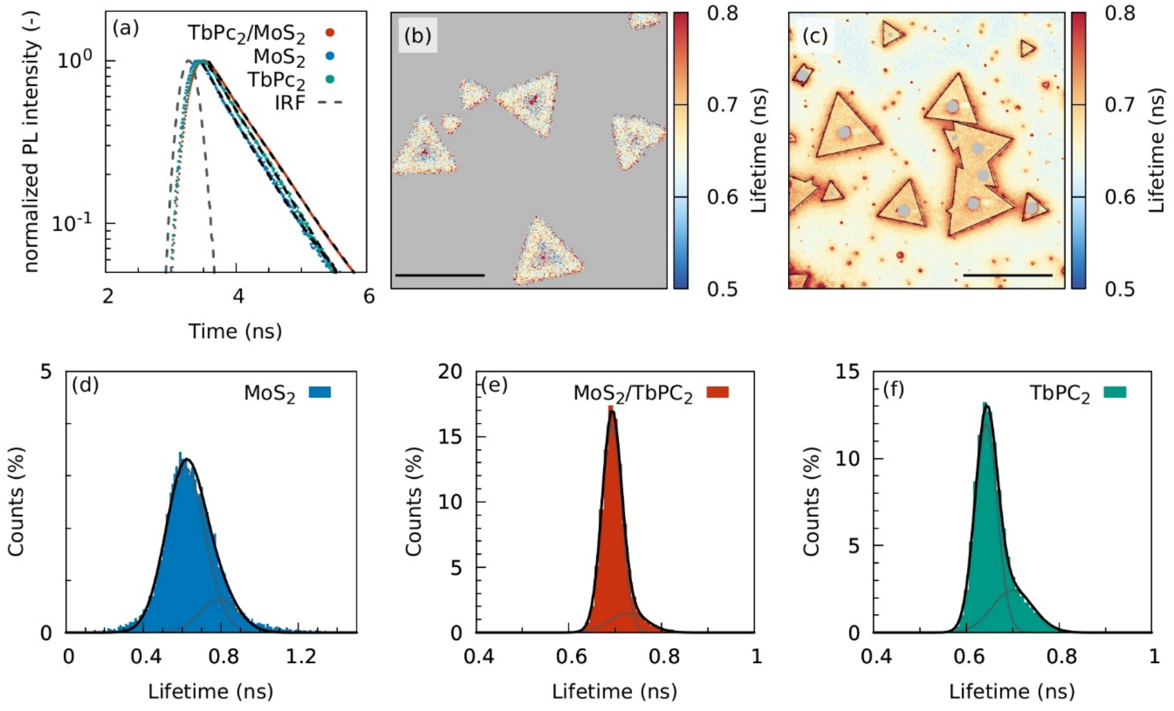

Figure S11. Results of the TRPL analysis of the (1L)MoS_2 layer, the $TbPc_2$ layer and the $TbPc_2$/(1L)MoS_2 spin hybrid structure. (a) TRPL decay profiles for the individual (1L)MoS_2 layer, $TbPc_2$ film and $TbPc_2$/(1L)MoS_2 heterostructure. The black dashed lines represent the exponential fit of the decay profiles, and the colored dots are the measured data. The grey dashed line shows the fitted instrument response function (IRF). (b) and (c) show the spatially resolved PL lifetime of (1L)MoS_2 and $TbPc_2$/(1L)MoS_2, respectively. The scale bar is 50 µm, and the grey circles in (c) correspond to the removed contribution of the few layer (1L)MoS_2 at the center of the flakes. The obtained lifetime histograms for (d) (1L)MoS_2, (e) $TbPc_2$/(1L)MoS_2 (area within the triangles in (c)) and (f) $TbPc_2$ film (area excluding the triangles in (c))

S3. Chemical vapor deposition of (1L)MoS_2

The MoS_2 monolayers were synthesized on SiO_2/Si substrate by one-step chemical vapor deposition (CVD) method in a horizontal single-zone at atmospheric pressure. A scheme of the CVD system is shown in Figure S12. The apparatus mainly consists of the heating zone and a quartz tube with a 60 mm diameter connected to the high-purity argon cylinder exhausted through deionized water. A small quartz tube with a diameter of 15 mm was employed to hold precursors and substrates together for the growth.

1×3 cm size of the SiO_2(300 nm)/Si wafer was used as substrate, further ultra-sonicated in acetone and iso-propyl alcohol in water bath for 10 min each and dried using ultra-pure argon gas blowing. The substrate was placed "face-down" on to a quartz crucible, containing 36 mg of MoO_2 and ~1 mg of NaCl distributed evenly which was then loaded into the small quartz tube, ensuring the location at proper heating zone. Subsequently, 82 mg of sulfur was introduced at the edge of the small quartz tube about 20 cm away from the crucible.

Before heating, the big quartz tube loaded with small quartz tube was purged with 120 sccm argon at atmospheric pressure for 15 min. The furnace was first heated from room temperature to 800 ^oC with a heating rate of 40 ^oC/min to obtain high nucleation points, ensuring that the sulfur portion remain outside furnace. The growth of MoS_2 was stimulated by moving quartz tube and introducing sulfur into furnace at about 750 ^oC for 10 min. The argon flow was maintained throughout the reaction. After growth, the furnace was allowed to cool down slowly to room temperature. A typical optical image of the sample is shown in Figure S13.

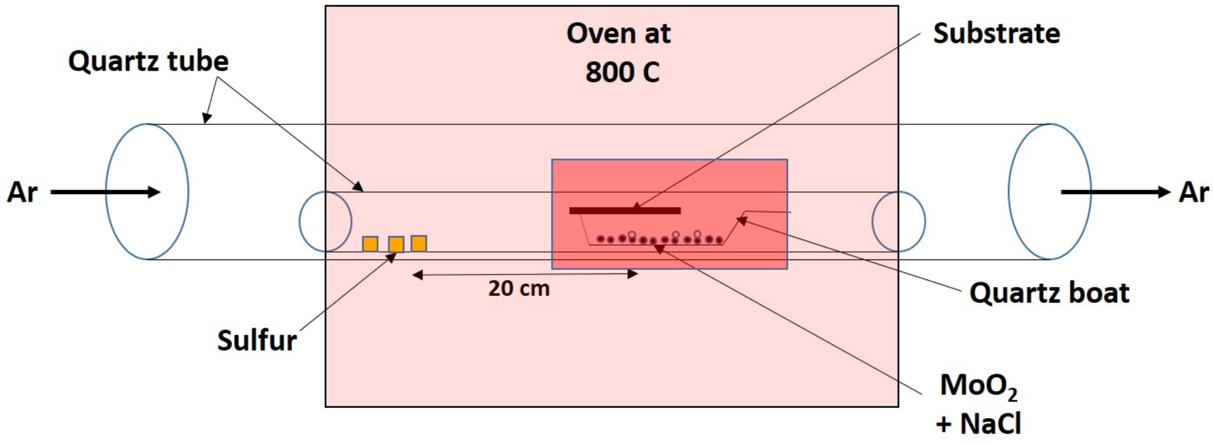

Figure S12. Scheme of the experimental set up for CVD of MoS_2.

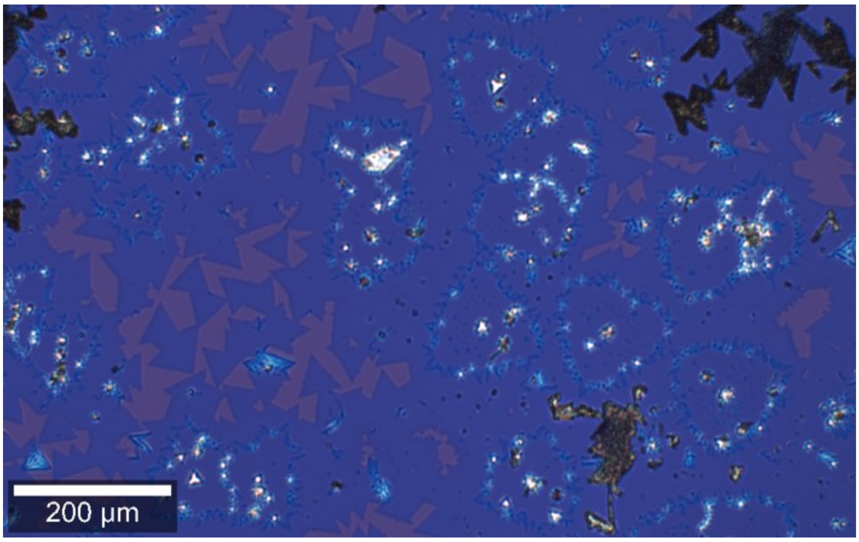

Figure S13. A representative optical image of (1L)MoS_2 monolayers grown by CVD.

References

[1] Amani, M.; Lien, D. H.; Kiriya, D.; Xiao, J.; Azcatl, A.; Noh, J.; Madhvapathy, S. R.; Addou, R.; Santosh, K. C.; Dubey, M.; Cho, K.; Wallace, R. M.; Lee, S. C.; He, J. H.; Ager, J. W.; Zhang, X.; Yablonovitch, E.; Javey, A. Near-Unity Photoluminescence Quantum Yield in MoS2. *Science (1979)* **2015**, *350* (6264), 1065–1068. https://doi.org/10.1126/science.aad2114.
